# Benzoylphenyl ureas as veterinary antiparasitics. An overview and outlook with emphasis on efficacy, usage and resistance

**DOI:** 10.1051/parasite/2019026

**Published:** 2019-05-01

**Authors:** Pablo Junquera, Barry Hosking, Marta Gameiro, Alicia Macdonald

**Affiliations:** 1 Vetparcs GmbH Zürich 8044 Switzerland; 2 Elanco Australasia Pty. Limited Kemps Creek 2178 NSW Australia; 3 Elanco Canada Limited 150 Research Lane, Suite 120 Guelph ON N1G 4T2 Canada

**Keywords:** Diflubenzuron, Fluazuron, Lufenuron, Novaluron, Teflubenzuron, Triflumuron

## Abstract

Six benzoylphenyl ureas are currently used in formulations approved as veterinary medicines: diflubenzuron for fly control mainly on cattle, lice and blowfly strike control on sheep, and lice control on farmed salmonids; lufenuron for flea control on dogs and cats and for lice control on farmed salmonids; triflumuron for lice and blowfly strike control on sheep; fluazuron for tick control on cattle; teflubenzuron for lice control on farmed salmon; and novaluron for fly and tick control on cattle and for flea control on dogs. Resistance to diflubenzuron and triflumuron has already been reported for sheep body lice and blowflies, and to fluazuron in cattle ticks. These and other minor veterinary usages, as well as the current status of resistance, are reviewed and perspectives for future opportunities are discussed based on unexplored potentials and threats posed by future resistance development.

## Abstract (fr)

Six benzoylphenyl urées sont actuellement utilisées dans des formulations approuvées comme médicaments vétérinaires : diflubenzuron contre les mouches des bovins, les poux et les myiases par calliphorides des ovins, et les poux dans le saumon d’élevage ; lufenuron contre les puces des chiens et chats et contre les poux dans le saumon d’élevage ; triflumuron contre les poux et les myiases par calliphorides des ovins ; fluazuron contre les tiques des bovins ; teflubenzuron contre les poux dans le saumon d’élevage ; et novaluron contre les mouches et les tiques des bovins, et contre les puces des chiens et chats. La résistance au diflubenzuron et triflumuron a déjà été rapportée pour le pou et les calliphorides des moutons, et au fluazuron pour les tiques des bovins. Le présent article résume ces usages vétérinaires et d’autres utilisations mineures ainsi que le développement de résistance, et discute les perspectives d’usage futur sur la base des potentiels non exploités et des menaces dues au développement future de résistance.

## Introduction

The disrupting effect of benzoylphenyl ureas (BPU) on the development of arthropods and their potential to control numerous pests was discovered in the early 1970s. Diflubenzuron was the first compound developed and subsequently numerous analogues of diflubenzuron have been discovered and introduced for the control of many arthropod pests in agriculture, forestry, public and private hygiene, and also animal health. Soon after their discovery, the common molecular mechanism of action disturbing chitin synthesis in arthropods was extensively investigated and progressively revealed [130, 199]; this resulted in their allocation to the so-called chitin synthesis inhibitors (CSI). Prior to the discovery of BPUs, other insect growth disruptors (IGD) were already known, mainly analogues of ecdysone and juvenile hormone, which do not interfere with chitin synthesis. Compared with most other pesticides, BPUs, together with all other IGDs have the significant advantage that they act upon arthropod-specific physiological and biochemical mechanisms that are absent in vertebrates, which makes them virtually non-toxic to mammals, birds, reptiles, amphibians and fish [274]. In contrast with this, most other pesticides act on the nervous system and are therefore more toxic to arthropod parasites, vertebrate hosts and operators. The major disadvantage of BPUs is that they are ineffective against the adult stages that, in most cases, cause damage to the host and are thus usually unsuitable for therapeutic use against established infestations. However, as shown in this review they have been successfully used as preventatives against a number of important veterinary pests.

The mode of action of BPUs and other CSIs has been reviewed recently [203]. Numerous studies with various BPUs have shown that they disturb cuticle formation at different levels that result in abortive molting and hatching defects in many insect orders. Ultrastructural analysis showed abnormal deposition of procuticular layers in response to treatment with BPUs. Studies in *Drosophila* larva showed that the cuticular phenotype induced by BPU treatment resembles that observed for embryonic mutants defective in the kkv gene encoding chitin synthase 1. Later on, several studies demonstrated that diflubenzuron efficiently blocks the incorporation of radiolabeled N-acetylglucosamine, the monomer of the polysaccharide *chitin*. However, the specific mechanism at the molecular level that leads to these effects has not yet been elucidated [203].

The literature on IGDs is extensive and the topic has been reviewed repeatedly (e.g. [79, 80]). The same applies to CSIs [203]. There are also numerous reviews focusing on BPUs (e.g. [274, 292]). However, most of these papers focus on the agricultural uses of BPUs and consider more or less extensively forestry and household pests, disease vectors, and veterinary parasites. Unfortunately, regarding veterinary parasites, these reviews have often been fragmented, incomplete and mostly out of the specific veterinary context. In fact, the use of BPUs as veterinary medicines has not been reviewed comprehensively. To our knowledge, one short overview has been published on the use of IGDs in animal health [125], but without a specific focus on BPUs. In the present publication, we review the use of BPUs as veterinary medicines for the control of veterinary parasites, i.e. applied on-animal, not in their environment.

Several BPUs have been investigated and are used against important vectors of human and veterinary diseases (e.g. mosquitoes, sandflies, black flies) that are also veterinary parasites [230, 243]. However, the control of these vectors is mostly not achieved through medications administered to the affected animals, but through treatment of the vectors’ environment, mostly aquatic. For this reason, they are not included in this review.

Six different BPUs have been introduced so far for the control of veterinary parasites, i.e. approved by regulatory authorities in major animal health markets: diflubenzuron, fluazuron, lufenuron, novaluron, teflubenzuron and triflumuron (Table 1). Whereas diflubenzuron, novaluron, teflubenzuron and triflumuron were developed first for plant protection and only later for veterinary uses, fluazuron was developed exclusively for veterinary use, and lufenuron was simultaneously developed for both purposes. Approved uses of BPUs include medicines registered for treating food producing animals, horses, cats and dogs. The efficacy of BPUs has also been investigated in several non-approved uses, or against parasites of minor domestic or wildlife species. The present review also reports on these minor uses documented in the scientific literature.

**Table 1.** Benzoylphenyl ureas currently approved for use on domestic animals.

Active Ingredient	Target pests	Target animals	Introduction	Major countries
Diflubenzuron	Dung-breeding flies	Cattle	1970s	USA
	Blowfly strike	Sheep	1990s	Australia, New Zealand
	Sea lice	Salmonids	1990s	Chile, Norway, Faroe Islands
Fluazuron	Ticks	Cattle	1990s	Australia, Latin America, South Africa
Lufenuron	Fleas	Dogs, cats	1980s	Worldwide
	Sea Lice	Salmonids	2010s	Chile
Novaluron	Fleas	Dogs, cats	2010s	USA
	Dung-breeding flies, ticks	Cattle	2010s	Brazil
Teflubenzuron	Sea Lice	Salmonids	1990s	Canada, EU, Norway, Faroe Islands
Triflumuron	Blowfly strike, lice	Sheep	1990s	Australia (lice), New Zealand

**Figure 1.**
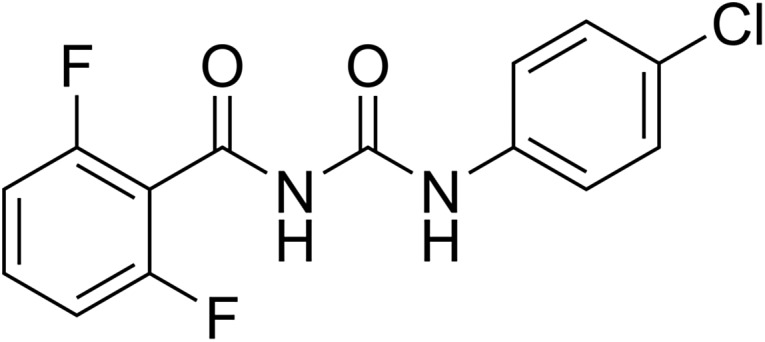
Chemical structure of difubenzuron.

Diflubenzuron (syn. TH 6040, PH 60-40) (Fig. 1) was the first BPU discovered and introduced as a pesticide by Philips-Duphar in 1975 under the trade name Dimilin^®^ [279]. Since then, hundreds of investigations have been carried out to study its efficacy against many agricultural, forestry and household pests, against vectors of public and veterinary importance, and against veterinary parasites. The scientific literature on diflubenzuron for pest and parasite control has been reviewed several times (e.g. [230, 243]). The first major commercial use of diflubenzuron as a veterinary medicine was against dung-breeding flies, mainly in cattle in the USA (Vigilante^®^ slow-release bolus from Duphar). About 20 years later, it was also approved for use against lice and blowfly strike in sheep (Fleececare^®^ from Hoechst, dipping and jetting fluid), and against sea lice in industrial aquaculture (e.g. Releeze vet^®^ from Ewos, a medicated feed product; CaliShot^®^ from FAV S.A., a premix to be mixed with the feed).

**Figure 2.**
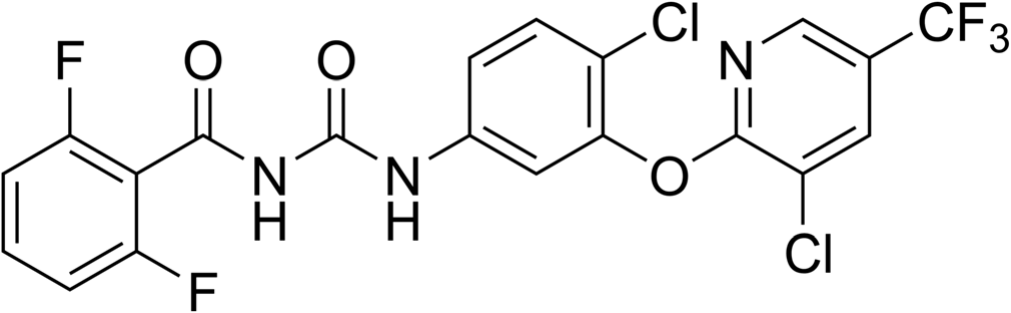
Chemical structure of fluazuron.

Fluazuron (syn. CGA 157419) (Fig. 2) was discovered by Ciba-Geigy and introduced in the animal health market in 1994 [160]. Acatak™ (now from Elanco), a ready-to-use pour-on, was launched first in Australia and Brazil. It was subsequently introduced in most Latin American countries and South Africa. Acatak is highly specific for the control and prevention of tick infestations on cattle, mainly one-host ticks such as *Rhipicephalus (Boophilus) microplus,* the southern cattle tick, *R. decoloratus*, the tropical cattle tick, and *R. annulatus*, the blue cattle tick. An injectable formulation had been investigated previously [53] but was abandoned. Fluazuron is not used in agriculture, private or public hygiene; it is exclusively used on cattle.

**Figure 3.**
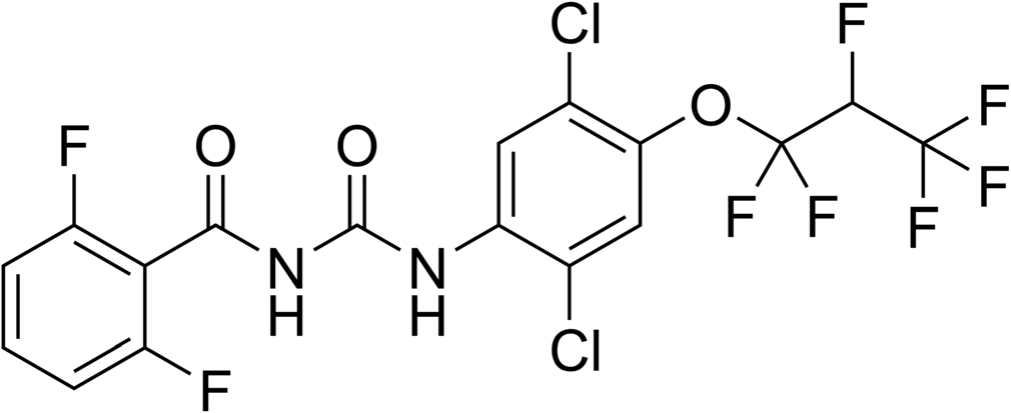
Chemical structure of lufenuron.

Lufenuron (syn. CGA 184699) (Fig. 3) was discovered by Ciba-Geigy in the mid-1980s and introduced around 1990 [279] almost simultaneously for flea control in dogs (Program^®^, now from Elanco) and as a crop pesticide (Match^®^, now from Syngenta). Program was the first once-a-month pill against fleas on dogs introduced in the pet market. Additional lufenuron formulations were launched for use in cats. It is now used in pets in many countries of the world. In 2016, lufenuron was approved as a premix formulation for oral administration via medicated feed, for sea lice control on farmed salmonids in Chile (Imvixa™, from Elanco). Besides its initially discovered efficacy as an IGD, some antimycotic properties against dermatophytes on mammals were reported (e.g. [81, 156, 257]), but no lufenuron-based veterinary product with antimycotic label claims has been developed so far. Like most BPUs, lufenuron is highly active against larvae of many insect species, but has almost no effect on adults and shows no efficacy against mites and ticks at practicable concentrations. A recent study has shown a certain effect of lufenuron on engorged females, eggs and larvae of *Rhipicephalus* (*Boophilus*) *annulatus* ticks when applied topically at high concentrations, but it basically confirms its unsuitability for tick control [3].

**Figure 4.**
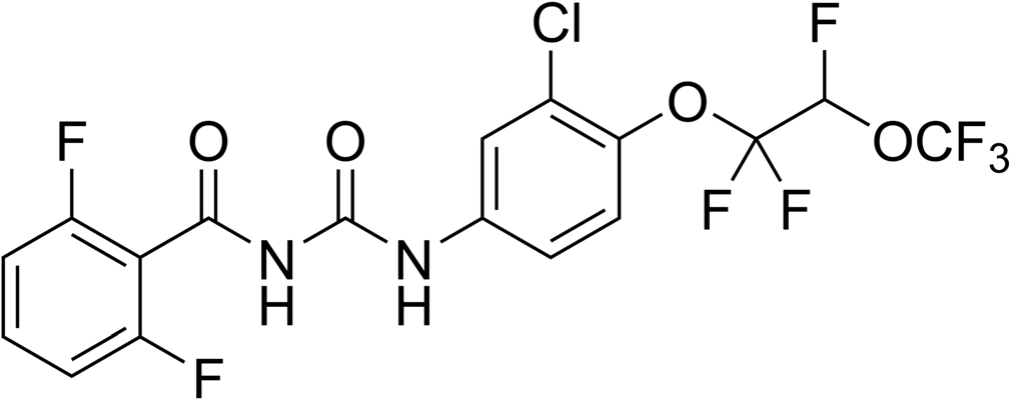
Chemical structure of novaluron.

Novaluron (Fig. 4) is one of the latest BPUs introduced. It was developed by Isagro and is marketed by Makhteshim [279]. It was approved for various crop protection uses in the United States (USA) in 2003, but not in the European Union (EU). Recently, it has been introduced in a few veterinary products for flea control in pets in the USA, in combination with fipronil (PetArmor^®^ plus IGR and Sentry^®^ Fiproguard^®^ plus IGR, both from Sergeant’s) and for cattle tick control in Brazil in combination with eprinomectin (Novatack^®^ Gold from Clarion).

**Figure 5.**
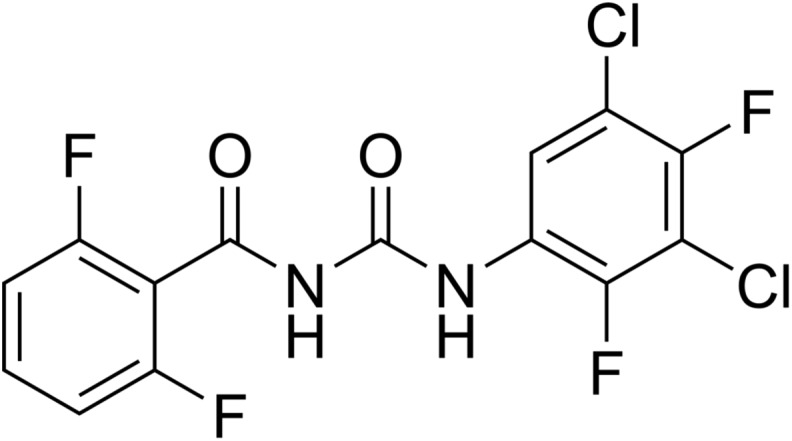
Chemical structure of teflubenzuron.

Teflubenzuron (syn. CME 134) (Fig. 5) was introduced in 1984 by Celamerck in Thailand for use against agricultural pests [279]. It has not been used on domestic animals, but it was introduced as Calicide^®^ in Norway in 1996 for oral administration in salmon against sea lice by Trouw [78, 248], and subsequently in other countries.

**Figure 6.**
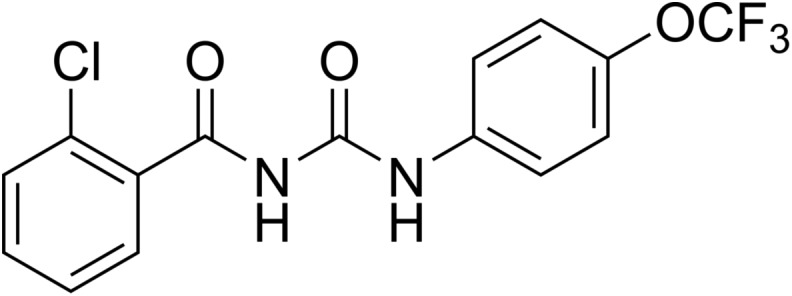
Chemical structure of triflumuron.

Triflumuron (syn. SIR 8514, VIR 7533) (Fig. 6) was discovered by Bayer and introduced for crop protection use (Alsystin^®^) in the early 1980s [112, 279]. Triflumuron was also developed for use in public health (Baycidal^®^) as well as for off-animal use in and around animal houses (Starycide^®^) against a number of flying and crawling insects such as houseflies and other filth flies, fleas, cockroaches, mosquitoes, etc. Triflumuron was first approved as a veterinary parasiticide in 1993 in Australia (Zapp^®^, from Bayer) as a ready-to-use pour-on for use on sheep against the sheep body louse, *Bovicola ovis* [141]. Veterinary parasiticides containing triflumuron are approved in Australia and New Zealand.

## Control of dung breeding flies in cattle and other domestic animals

There are two major groups of muscoid flies that breed in fresh dung or in organic waste abundant in livestock facilities those flies that feed directly on the animals and are obligate parasites, and those filth and nuisance flies that may or may not feed on the animals but are important vectors of diseases that can contribute to serious hygiene problems in livestock operations. Among the obligate parasites, the most important species are horn flies that feed on blood mainly from cattle (*Haematobia irritans irritans* in Europe, America, Northern Africa and parts of Asia; *H. irritans exigua*, the buffalo fly in Australia and parts of Asia; *H. thirouxi potans*, the African buffalo fly in most of sub-Saharan Africa) and stable flies (*Stomoxys calcitrans*) that feed on blood on all kinds of livestock worldwide. Adult face flies (*Musca autumnalis*) are found mainly in Europe, Asia, North Africa and North America and are not hematophagous but feed on body fluids (around the eyes, the nostrils, etc.) mainly from cattle. The most important and cosmopolitan species among the non-hematophagous flies is the housefly, *M. domestica* abundant in all livestock operations. Lesser houseflies (*Fannia canicularis*), false stable flies (*Muscina stabulans*), black garbage or dump flies (*Ophyra* spp.) and flesh flies (*Sarcophaga* spp.) are other fly species that can become a problem in livestock operations, particularly in and around dairy, pig and poultry facilities [177].

These flies lay their eggs on dung or on organic waste abundantly found in any livestock production environment. Larvae hatch out of the eggs, feed on dung or other organic waste, and complete development to pupae and adult flies within a few days or weeks, depending on the species and climatic conditions (mainly temperature and/or humidity). In most regions, these flies show seasonal development, with peaks during the warm and/or rainy season. Horn flies and face flies breed on fresh cowpats and are a problem mainly for cattle kept on pasture at low densities that allow most of the cowpats to dry out undisturbed. Stable flies breed on decaying vegetable material, ideally on any kind of animal dung mixed with straw or hay and kept humid with urine, as typically found in feedlots, and in and around dairy and pig farms. Houseflies and other nuisance flies are capable of breeding on any decaying organic material, whereby large manure accumulations as often found in cattle feedlots or in dairy, pig and poultry farms (particularly in layers) are very favorable for breeding of these flies [177].

Diflubenzuron is effective mainly against the eggs and larvae of all these flies, not against the adults. For several species it has been shown that if the adult flies are directly treated, enough diflubenzuron will be deposited in their eggs to inhibit larval hatch or their subsequent development to adult flies. If the breeding medium (dung, manure, waste, etc.) is treated with diflubenzuron, larvae will ingest it and will not complete development to adults [230, 243].

Two major approaches have been followed to control dung or waste-breeding flies in livestock production using diflubenzuron: on-animal and off-animal use. On-animal use consists mainly in oral administration of diflubenzuron to livestock to ensure that all manure produced contains enough quantities of active ingredient for inhibiting larval development. Oral administration is done either admixing diflubenzuron to the food or mineral salts, or in the form of slow-release boli. Topical administration (spraying and dusting) has also been investigated. Off-animal use consists mainly in directly treating manure or organic waste (so-called “larviciding”) with diflubenzuron. These two approaches to fly control in livestock production have been practiced with other pesticides as well, e.g. with methoprene and cyromazine, two other IGDs, or with some organophosphate larvicides [12, 13, 177].

### Efficacy

In one of the first studies published on this topic, the efficacy of diflubenzuron against face flies (*M. autumnalis*) and houseflies (*M. domestica*) was investigated in dairy cows fed with concentrate mixed with diflubenzuron to achieve doses of 0.25, 0.5, 1, 5 and 10 mg/kg body weight (bw). Manure samples were periodically collected and seeded with larvae of laboratory-reared flies. The percentage reduction of adult fly emergence from seeded face fly larvae was about 90% and >99% in manure of cows treated at 0.25 and 0.5 mg/kg, respectively. For houseflies, the percentage reduction was about 50% and >95% in the manure of cows treated at 0.25 and 0.5 mg/kg, respectively. At higher doses, 100% reduction was achieved [207]. In another experiment, mineral blocks containing 0.1 or 0.05% diflubenzuron were offered to individual steers in confined pens for 30 days. The blocks were weighed periodically to determine intake. Fresh manure was collected daily from each pen and samples were seeded with laboratory-reared housefly eggs. Exposure to the mineral blocks containing 0.1 and 0.05% diflubenzuron caused a maximum of 98% (days 27–33) or 91% (days 21–26) reduction of adult hatch in the corresponding manure samples [290]. High efficacy against horn flies (*H. irritans irritans*) was also reported in a study after providing mineral blocks containing 0.1 and 0.05% diflubenzuron to cattle for nine weeks. Manure samples of treated cattle were collected periodically. They were observed for emergence of adult flies and bioassayed with horn fly larvae. Inhibition of adult fly emergence in the bioassays was 75 and 83% in manure of cattle that consumed the 0.05 and 0.1% mineral blocks, respectively [14]. More recent studies using commercial formulations of diflubenzuron approved for use on cattle in the USA (Clarifly^®^, Wellmark International) confirm the efficacy of feed-through medication of feedlot and dairy cattle with diflubenzuron for controlling dung-breeding flies [7]. In a more recent field study in Brazil, the efficacy of diflubenzuron added to mineral salts was investigated for the control of horn flies. For five months, a group of 20 cattle received mineral salt *ad libitum* that had previously been medicated with diflubenzuron (Difly^®^ 25%) at a dose of 0.5 g/kg mineral salt. Average salt consumption by cattle was about 70 g/animal/day. Manure samples were periodically collected and seeded with horn fly eggs to determine the emergence of adult flies in the laboratory. Adult flies on cattle were also counted periodically in the field. Average emergence of adult flies in manure samples of untreated cattle was 86%, but only 1% in treated cattle. The number of adult flies counted on treated cattle was reduced by 99% [77].

Triflumuron was also investigated as a feed-through medication for cattle at rates of 0.016 and 0.125 mg/kg mixed into feed concentrate. It resulted in >99% control of larvae of face flies and houseflies seeded into the manure [208], but no commercial product was developed with this active ingredient.

Recently, novaluron was investigated in cattle as a feed-through larvicide against horn flies (*H. irritans irritans*), stable flies (*S. calcitrans*), and house flies (*M. domestica*) [188]. Using a non-commercial formulation containing 0.67% novaluron, a group of Hereford calves was treated at rates of 0.4 and 0.6 mg/kg bw/d for 10 days. The daily dose was administered orally within gelatin capsules using a balling gun. Fresh manure was collected daily from treated and untreated control animals for 14 days following treatment. The manure samples were bioassayed with eggs of laboratory strains of the mentioned fly species. Novaluron resulted in 100% reduction of adult stable fly emergence at both rates over the whole period, starting on the second day after treatment. Emergence of horn fly adults was reduced by ~60% at 0.4 mg/kg, and ~95% at 0.6 mg/kg. Emergence of house fly adults was reduced by ~80% at 0.4 mg/kg and by ~90% at 0.6 mg/kg. The level of control diminished from day 9 after treatment.

The efficacy of diflubenzuron was also investigated after topical application to cattle, i.e. directly exposing the adult horn flies to diflubenzuron when visiting their hosts for blood-feeding. In a laboratory trial, diflubenzuron was applied as a dust to cattle housed individually in enclosed stalls at rates of 3 or 6 mg per animal. Laboratory reared flies were released onto the treated animals and their eggs were collected to determine whether they developed or not. At 3 mg diflubenzuron/animal, the yield of adult horn flies was reduced by 86%. At 6 mg/animal, no adult horn flies were produced. In two field studies, cattle were exposed to dust bags containing 0.5 or 1% diflubenzuron. In both studies, horn fly production was eliminated [173].

The effect of spraying cattle with diflubenzuron on the reproductive performance of horn flies landing and feeding on such treated animals has also been investigated. In one field study, cattle were sprayed with 0.5 or 1% diflubenzuron. Manure pats were collected before and after treatment and eggs found in the manure were reared to determine hatching. Eggs from laboratory flies were also seeded on manure from treated cattle. The 1% spray treatment resulted in complete elimination of adult fly emergence for four weeks after treatment. The authors suggest that licking may have been the major route for diflubenzuron to get into the animals’ manure [172].

The efficacy of a slow-release bolus containing diflubenzuron administered to cattle was investigated for the control of flies in the USA in the 1980s. In one study, differently manufactured 50 g boli (molded and compressed) containing 10% diflubenzuron were administered to test cattle, and manure samples were periodically bioassayed with laboratory-reared larvae of horn flies (*H. irritans irritans*), face flies (*M. autumnalis*), stable flies (*S. calcitrans*) and houseflies (*M. domestica*). Both molded and compressed boli provided 14–17 weeks control of horn and face flies. Control of stable flies and houseflies was less effective [205]. In a series of field studies on pastured cattle, commercial boli (Vigilante^®^; containing 10% diflubenzuron and administered at a rate of one bolus per 250 kg/bw) and various experimental boli (with variable diflubenzuron content) were administered to pastured cattle. Subsequently, the number of horn flies and face flies were counted on 10–15 animals every two or four weeks. Manure samples were also collected biweekly until 20 weeks post-treatment and bioassayed with larvae of face flies, horn flies and houseflies. In most herds, the boli achieved >80% control of face flies and horn flies in the manure of treated cattle [212]. In another series of field studies, the efficacy of the previously mentioned commercial bolus and other experimental boli was investigated on dairy farms. Flies were counted periodically on the cows and manure samples were taken for bioassays with larvae of horn flies, face flies, houseflies and stable flies. Bolus treatment resulted in >85% mortality of face fly larvae and 47 to >85% mortality of horn fly larvae. There was no indication that the populations of stable flies and houseflies on cattle were reduced by treatment [211]. In another study on cattle treated with the same commercial bolus, periodically collected samples of manure were bioassayed with horn fly larvae and showed a reduction in fly emergence of 82.9–100% over a period of 21 weeks, and no reduction at week 27 post-treatment [107].

The potential of diflubenzuron fed to pigs to inhibit the development of houseflies in the manure was investigated in one study. Two groups of four pigs kept in separated pig sties received feed containing diflubenzuron to ensure a dose rate of 0.25 or 0.5 mg/kg bw/day for three weeks. Manure was periodically collected and seeded with larvae of laboratory-reared houseflies (*M. domestica*). Feeding at 0.5 mg/kg/day resulted in almost complete inhibition of adult hatching during the three weeks after start of the medication. Inhibition strongly declined in manure collected four days after ending the medication. At the lower dose rate of 0.25 mg/kg/day, a maximum inhibition of hatch of about 97% was achieved during the third week [244]. In another study at a commercial pig farm, the animals received feed containing diflubenzuron to ensure a dose of 0.5 or 1.0 mg/kg/day over 115 and 71 days, respectively. Manure samples were periodically collected either for counting fly pupae, or to be seeded with larvae of laboratory reared houseflies (*M. domestica*) and bronze dump flies (*Ophyra aenescens*) to determine adult emergence. Adult flies were periodically counted in the facilities to estimate the impact of treatment on the fly population. Fly populations were reduced by 100% within 6–8 weeks after starting the medication. Emergence of adult flies in manure samples of medicated pigs was significantly reduced (92–100% depending on the time after treatment) but not completely eliminated [17].

The efficacy of diflubenzuron to control houseflies after feed-through medication administered to chickens was investigated soon after its discovery. In an experimental study with laying hens (White Leghorn, WL and New Hampshire, NH breeds), diflubenzuron mixed in the feed at a rate of 50 ppm was administered to the birds over a three-week period. Subsequently, the ratio was halved every three weeks until the final rate of diflubenzuron in the feed was 1.6 ppm. Manure samples were taken periodically and seeded with larvae of laboratory-reared houseflies. Eggs were also collected to determine diflubenzuron residues. Emergence of adult flies in the manure samples was completely inhibited in hens treated with feed containing 12.5 ppm or more diflubenzuron. At lower rates, emergence was only partially inhibited. Residues in the eggs reached between 1.2 and 2.9 ppm in hens that received medicated feed with 50 ppm diflubenzuron, down to 0.03–0.05 ppm in hens that received medicated feed with 1.6 ppm diflubenzuron [209]. In a subsequent study, WL hens and Black Sexlinked Cross hens (BS) received medicated feed containing 10 ppm diflubenzuron for 15 weeks. As in the previous study, manure was periodically collected for bioassays with housefly larvae or for residue determination. Emergence of adult houseflies in manure of treated hens was reduced by >95% [210]. In a later study in Brazil, hens received medicated feed containing diflubenzuron (2 g of diflubenzuron 25% per kg feed) for two weeks. Manure samples were collected and emerging adult flies were counted and determined. Reduction in the emergence of adult flies was almost 100% until about one week after medication was discontinued [263].

Triflumuron was also tested against flies breeding in manure or other organic waste. In laboratory tests, larvae of blood-sucking stable flies (*S. calcitrans*) reared in artificial larval medium previously treated with various amounts of triflumuron did not complete development to adult flies [171]. In one study as a feed-through in mini-broiler hens, approximately 19 ppm in the feed for 3–4 weeks was needed to achieve >95% mortality of housefly larvae seeded into the droppings [208]. In another study on WL and Vedette mini-broiler breeder hens, triflumuron added to the feed at 15–25 ppm for four weeks achieved >95% control of houseflies [213].

As can be expected, diflubenzuron and other IGDs act only on the non-parasitic immature stages of the flies, and adults that cause the damage or nuisance are not killed. This means that diflubenzuron or any other IGD do not knock-down fly populations within hours, as insecticides with an adulticidal effect usually do. Depending on temperature, humidity, access of flies to untreated materials and other extrinsic factors, it takes 2–3 weeks for the effect to become fully evident to users. IGD products are therefore particularly appropriate for preventative treatments. For indoor use in animal houses, this is often acceptable because there is little immigration of flies from outside. However, after outdoor use, e.g. against horn flies and face flies in cattle, control may be insufficient because flies from neighboring properties can easily re-infest the treated herds, since it is known that horn flies and face flies can fly over long distances [31, 174, 194].

### Commercial use

Feed-through administration of diflubenzuron for the control of dung-breeding flies in livestock and poultry was the first and quite extensively investigated usage of diflubenzuron for domestic animals. The slow-release bolus with diflubenzuron for cattle (Vigilante^®^, American Cyanamid, now Chemtura) was approved in the USA in the 1980s and has been used since then for the control of dung-breeding flies (mainly horn and face flies). To our knowledge, it has not been used in Australia, Canada, Europe, and most of Latin America, where horn flies are also a serious cattle pest. Diflubenzuron-based feed-through products to be added to food or mineral salts were approved in the USA only about a decade ago. They are available for use on cattle (e.g. Clarifly^®^, from Wellmark) and also on horses (e.g. Simplifly^®^, from Farnham). In Europe, a few feed-through brands were approved for use on livestock and poultry in the 1980s: Duphacyd^®^ (Salsbury-Solvay) in the UK for use on pig and poultry; and Astonex^®^ (Shell) in The Netherlands for use on cattle, pig and poultry [112, 265]. However, they are no longer available today. We have not found evidence that BPU-containing products for topical use (spraying, dusting, pour-on, etc.) have been marketed so far for the control of dung-breeding flies on domestic animals. To our knowledge, no other BPU is currently marketed for on-animal use against dung-breeding flies in livestock.

The poor global commercial use of diflubenzuron against dung-breeding flies is probably related to the fact that more immediately effective and therefore commercially more attractive alternatives are available in most regions, particularly insecticide-impregnated ear-tags and pour-ons with adulticidal effect, i.e. with rapid knock-down of fly infestations as well as demonstrated repellent effects. Although horn flies have developed high resistance to synthetic pyrethroids in many countries and to some extent also to organophosphates [100], they are still widely used and new active ingredients of newer chemical classes or mixtures that contribute to overcome resistance have been introduced in many countries. A recent survey on commercially available ectoparasiticides in Latin America showed that more than 300 commercial brands containing pyrethroids are used on livestock against flies and ticks, mainly in cattle [161]. No single veterinary medicine containing BPUs for fly control on livestock was identified in the survey.

## Flea control on dogs and cats

*Ctenocephalides felis* is the most abundant flea species infesting dogs and cats worldwide [19, 169] and most studies on flea control with lufenuron were conducted on this species.

Only adult fleas infest cats, dogs and other mammalian hosts. Immature stages always remain off the hosts in the environment. After their blood meal, adult female fleas lay eggs that fall to the ground. Larvae hatch out these eggs on the ground, where they molt several times and complete development to pupae. Adult fleas that hatch out of the pupae find a host where they feed on blood and reproduce. The whole life cycle can take between two weeks and more than three months, depending on temperature and humidity [86, 169].

### Efficacy

The efficacy of lufenuron orally administered to dogs and cats is based on lufenuron compartmentalizing into the host’s blood from where it is ingested by adult female fleas and subsequently deposited inside their eggs during oogenesis. At the therapeutic dose administered to dogs or cats (for oral administration usually 10 mg/kg for dogs and 30 mg/kg for cats), ingested lufenuron does not significantly control adult fleas on the treated pets, but their eggs are not viable: larvae die inside the egg envelopes [73, 85, 137, 293] or after hatching [72]. As a consequence, the life cycle is interrupted and the flea population in the environment is progressively decimated. Lufenuron has no adulticidal effect on adult fleas, i.e. it will not kill most adult fleas infesting a pet at the time of treatment. For this reason, it is recommended to use it preventatively starting at the beginning of the flea season, before hatching of the first fleas emerging after overwintering in the pet’s environment.

Besides the effect on the immature stages, it has been shown that lufenuron also affects adult fleas. In laboratory studies, after ingestion of blood containing 0.5–4 ppm lufenuron, adult fleas showed disturbances on the development of the endocuticle, likely to cause a decrease in resiliency of the cuticle to expansion during blood feeding. Inhibition of midgut epithelial cell differentiation was also observed in adult fleas after lufenuron ingestion. These effects were considered responsible for up to 24% mortality [74]. However, the lufenuron concentration at which mortality was observed in these experiments was significantly higher than expected in the blood of pets treated at the therapeutic dose.

Flea feces consist mainly of dried blood and are the main nutrient for developing flea larvae in the environment of the pets [86, 169]. The presence of lufenuron in flea feces was investigated in a laboratory study with cats previously treated with lufenuron and artificially infested with *C. felis*. The study concluded that there was a strong correlation between the lufenuron concentration in the feces and the mortality of larvae feeding upon such feces [293].

High preventative efficacy lasting for about one month after a single treatment and proportionally longer after repeated treatments has been shown *in vivo* after oral administration to experimentally infested dogs [20, 113, 114, 138, 220, 267] and cats [21, 22, 108, 220]. The efficacy of orally administered lufenuron achieved under controlled conditions was confirmed in several field studies [103].

After the introduction of lufenuron-containing tablets for dogs and an oral suspension for cats, a long-acting injectable formulation of lufenuron was developed exclusively for cats. A single injection at the recommended therapeutic dose (10 mg/kg) ensured >90% prevention of re-infestation for up to six months [115, 254].

Flea allergic dermatitis (FAD) is a serious condition that often affects dogs [109] and cats [56] as a consequence of hypersensitivity to flea salivary proteins. Effective control of fleas is an essential measure for FAD therapy. It was shown that flea control achieved by monthly lufenuron treatments substantially reduced the incidence of FAD in dogs and cats [106]. Nowadays flea control with lufenuron continues to be considered as an adequate option as part of FAD therapy in these animals [33, 57] and is included in the label claims of lufenuron-containing medicines in several countries.

Novaluron has recently been introduced for flea control in a few spot-ons for dogs combined with fipronil. Novaluron adds larvicidal efficacy to the adulticidal effect of fipronil. Many similar spot-ons contain other IGDs, mainly S-methoprene or pyriproxyfen. Although it can be assumed that novaluron ensures such an IGD effect against fleas, we have not found published data and rely on regulatory marketing authorizations as the evidence.

### Commercial use

Lufenuron was the first once-a-month tablet for flea control introduced in the pet market in the early 1990s (Program^®^, Ciba-Geigy, later Novartis, now Elanco) although it was not the first flea-control drug for oral administration. Oral medications for the control of fleas on dogs and cats were already available in a few countries before the introduction of lufenuron. Decaflea^®^ (from Dermacare-Vet) was available in Australia for dogs and cats and consisted in a combination of cyromazine (another IGD) and diethylcarbamazine citrate, and was primarily used for the prevention of heartworm infections after daily administration to dogs [260]. Several commercial brands containing cythioate, an organophosphate, were also available for dogs in several countries, (e.g. Proban^®^ and Cyflee^®^, from American Cyanamid in the USA and Germany, respectively, available as tablets to be administered orally twice a week) [265]. To our knowledge, these oral medications for dogs with cyromazine or cythioate have been discontinued.

For dogs, tablets containing both lufenuron and milbemycin oxime were subsequently introduced in the mid-1990s (Sentinel^®^ from Novartis) indicated for both flea and heartworm prevention and for the control of several parasitic worms of dogs. Finally, about a decade later, Novartis introduced a combination of lufenuron, milbemycin oxime, and praziquantel (Sentinel Spectrum^®^) that added efficacy against tapeworms.

The launch of Program in the USA was overwhelmingly successful and lufenuron quickly gained flea-control market leadership in many countries. However, a few years later very effective topical, non-systemic once-a-month spot-ons with efficacy against adult fleas were introduced: Frontline^®^ from Rhône-Mérieux (later Merial) with fipronil, and Advantage^®^ from Bayer with imidacloprid. Usage of lufenuron decreased significantly, most probably due to its lack of adulticidal effect on established flea infestations.

For about 20 years lufenuron-based products from Novartis (now Elanco) remained the only once-a-month oral medication for flea control on pets. Later, tablets containing spinosad (Comfortis^®^ from Elanco) were also introduced for dogs [268] and more recently several isoxazolines for oral administration to dogs and effective against both fleas and ticks have been introduced [88, 201, 288].

Nowadays, lufenuron remains available for flea control in many countries and use is moderate to low. Although patent protection of lufenuron expired about 10 years ago, other animal health multinationals and most well-known local or regional manufacturers of veterinary parasiticides have not developed their own brands containing lufenuron. In a recent survey (July 2017), we found two generic brands in Europe (Vlooien Anti-conceptie^®^ from Beaphar; Flee Fence^®^ from Diergeneesmiddelen, both in The Netherlands), four brands in Latin America (Sinpulgar^®^ from Chalver, Colombia; Lufenuron from Vetpharm, Brazil; Gets Plus from IDV and Spinomax dúo from Zoovet, both with lufenuron and spinosad, Argentina) and a few generic products of unknown origin offered by some online shops. In contrast with this, hundreds of generic brands are available worldwide containing fipronil, permethrin, imidacloprid or other generic active ingredients vastly used in topical flea control products for pets.

To our knowledge, novaluron is currently used only in a few out of dozens of spot-ons for dogs in the USA that have been recently introduced. So far, it is not used in Europe or Latin America. It can be assumed that usage is still very limited.

## Lice control on sheep and other domestic animals

Lice that affect sheep and other domestic animals spend their whole life on their host, i.e. all developmental stages from egg to adults live in the fleece of affected sheep. There are two major groups of lice that affect domestic animals: hematophagous lice, also called sucking lice (Anoplura) and non-hematophagous lice, also called biting or preferably chewing lice (Mallophaga). All louse species of domestic animals are very host-specific [177]. Lice are of particular concern for the sheep industry, especially the sheep body louse, *B. ovis* (Phthiraptera, Trichodectidae; formerly *Damalinia ovis*). Although not very pathogenic to sheep, they cause considerable economic losses due to reduced wool production (up to 1 kg/sheep), poor quality wool (cotted, yellow) and damaged hides [151, 154, 158]. Lice affect sheep and other domestic animals worldwide, but they have particular economic importance for the sheep industries in Australia and to a lesser extent in New Zealand.

Like other louse species *B. ovis* is very host specific, i.e. it won’t infest other livestock. It is a rather small (<2 mm) non-hematophagous chewing louse that feeds on skin debris, lipid and gland secretions, superficial skin cells, skin bacteria, etc. The life cycle is usually completed in 34–36 days. The lifespan is usually 28 days (maximum of 53) for adult females, 49 days (maximum 74) for adult males. Transmission of lice is through contact among sheep, particularly when crowded during yarding, housing, etc. [151, 154, 158].

### Efficacy

Two BPUs have been successfully used for the control of sheep body lice, triflumuron and diflubenzuron. Efficacy of both compounds against *B. ovis* or other sheep lice species is very poorly documented in the literature. We have not found scientific papers on this topic. A condition for marketing authorization of sheep lousicides in Australia is 100% efficacy against natural infestations of *B. ovis* proven in field studies (when animals are treated off-shears, i.e. within the first 24 h after shearing, or with <six weeks wool growth); consequently, all manufacturers of approved products have provided the corresponding study reports to the registration authorities, but these reports are not publicly available. Summarized information on the efficacy of diflubenzuron in field studies is reported in the technical manuals of some commercial products for topical administration. In one such technical manual (Coopers^®^ Magnum^®^), it is reported from six field studies in which, after off-shears topical treatment with a pour-on formulation containing 2.5% diflubenzuron, the reduction in louse numbers was >99% six weeks after treatment, and 100% 12 and 20 weeks after treatment. Most common commercial products containing triflumuron are ready-to-use pour-ons for topical application containing 25 g/L active ingredient. According to product labels (e.g. from Zapp Pour-on, Bayer) the recommended dose is 1 mL/kg bw (i.e. 25 mg/kg) for animals up to 10 kg and decreases progressively with the animal’s weight, down to a minimum of about 0.37 mL/kg (i.e. about 9.25 mg/kg) for animals over 85 kg bw. Dosing is based on body surface and not on body weight. Administered at this dose to sheep, it prevents the development of immature stages for the following 20 weeks. Treated sheep are protected against re-infestation with body lice for 12 weeks.

Efficacy of diflubenzuron, triflumuron and other BPUs against louse species of other domestic animals has been reported in the literature. Efficacy of diflubenzuron against lice was reported first in the USA on Angora goats affected by the goat chewing louse (*B. limbatus*). More than 90% control was achieved for up to 14 weeks after spray-treatment of the animals three weeks post-shearing with 0.1 and 0.2% diflubenzuron [46]. In later studies, pour-on treatment (30 mL of 2% diflubenzuron as a suspension in water) six weeks after shearing completely eliminated goat louse infestations for up to 18 weeks after treatment [204]. In a study in South Africa, goats naturally infested with the chewing louse (*D. limbata* syn. *B. limbatus*) were dipped in diflubenzuron at a concentration of 625 ppm and subsequently kept in quarantine. Lice were completely eliminated by week 6 after treatment. In goats returned to the main flock after treatment, average percentage control for 24 weeks was 84–88% for nymphs and adults, respectively [111].

Natural infestations of horses with the chewing louse *Werneckiella equi* (formerly *Damalinia equi*) were successfully controlled using a commercial triflumuron pour-on formulation approved for louse control on sheep administered at a dose of 2.5 mg/kg bw (1 mL/10 kg bw). Efficacy of 100% was achieved in two separate studies 44 and 71 days after treatment [190]. In another report, the efficacy of diflubenzuron against the horse chewing louse, *W. equi*, was studied in ponies. Five naturally infested Shetland ponies were treated with a pour-on containing 5% diflubenzuron and 5% permethrin (3 mL/45 kg bw). Six weeks after treatment no lice were found on the treated animals [242]. In a study in the USA on the efficacy of several active ingredients and formulations against cattle naturally infested with lice (mixed infections with *B. bovis*, *Haematopinus eurysternus*, *Linognathus vituli* and *Solenopotes capillatus*), diflubenzuron applied topically as a pour-on (3%) achieved 99% control of lice at week 6 after treatment and 100% control eight weeks after treatment [36].

### Commercial use

BPUs for louse control in sheep have been highly successful in Australia and New Zealand. Soon after the introduction of the two first brands (Zapp from Bayer with triflumuron; Fleececare from Hoechst with diflubenzuron), the BPUs dominated the lousicide market in Australia [286]. This was partly driven by increasing parasite resistance to synthetic pyrethroids, the previous market leading class, and to the quick decline of organophosphate use [249]. In the meantime, and after patent expiry, numerous other triflumuron brands for use on sheep were approved in both countries, mostly as pour-ons. In New Zealand, concentrates for jetting (containing 480 g/L) and mixtures (e.g. with cypermethrin or imidacloprid) have been approved. In 2010, out of 25 sheep lousicide brands approved in Australia, eight contained triflumuron (mainly 2.5% pour-ons), five diflubenzuron, four cypermethrin, three ivermectin, two spinosad, and one each contained abamectin, diazinon and imidacloprid [159]. There are two lousicides containing a mixture of diflubenzuron and dicyclanil (e.g. CLiK Plus™ from Elanco). In New Zealand, a recent search (2017) in the online database of the Ministry of Primary Industries yielded nine brands containing triflumuron and eight brands containing diflubenzuron approved for use on sheep.

A few brands containing BPUs are also approved for use as lousicides on cattle and/or horses, e.g. Clean-up^®^ from Bayer, a pour-on containing 5% permethrin and 5% diflubenzuron approved in the USA for cattle and horses; Lice “n” Simple^®^ from Jurox, a pour-on containing 2.5% triflumuron approved for use on horses in Australia and New Zealand. However, these are only minor uses compared with lice control on sheep.

## Blowfly strike control in sheep

Blowflies (Diptera, Calliphoridae) cause extensive cutaneous myiasis on sheep, so-called blowfly strike. The most damaging species are *Lucilia cuprina* found mainly in Australia, New Zealand, North America, and South Africa, and *L. sericata*, found mainly in Europe and New Zealand. Both species lay their eggs on the surface of the sheep’s fleece. Odors associated with putrefaction, fleece rot bacteria or dermatophilosis are particularly attractive for ovipositing females. Larvae (~1 mm long) hatch within a few hours and crawl down the fleece to the skin surface. They are not capable of piercing the host’s skin but feed on skin debris, bacteria, and exudates around small wounds, etc. Within a few hours, they molt to larger second instar larvae that can already abrade the skin, which then molt to larger third instar larvae that feed voraciously on the host’s skin and underlying tissues and can reach up to 1.5 cm length. As they feed, the initially small skin injuries become larger and deeper. They attract more ovipositing flies and the injuries become infected with secondary bacteria. Left untreated, sheep may die in a few days. Mature larvae fall to the ground where they pupate. Adult flies are not parasitic to sheep but need one protein meal to lay eggs. The life cycle can be completed in 12 days under ideal conditions. Both species are not sheep-specific parasites and can also develop on wounds, necrotic tissues, and any type of decaying carrion, carcasses, etc. Blowflies are a very serious pest, mainly in Australia, Ireland, New Zealand, South Africa and the UK, causing substantial losses to the sheep industry [117, 180].

### Efficacy

Efficacy of diflubenzuron against larvae of *L. cuprina* was reported first from laboratory tests that confirmed its mode of action as a CSI in this species [281]. In subsequent laboratory studies, treatment of adult *L. cuprina* flies with diflubenzuron completely inhibited hatching of their eggs both after topical treatment [184] and mixed with the fly food [185]. The suitability of diflubenzuron to protect sheep against blowfly strike caused by *L. cuprina* was investigated later in an indoor study under controlled conditions. In a first experiment under moderate fly pressure, sheep jetted with diflubenzuron at 1000 ppm were protected against flystrike for at least 110 days. At 1500 ppm protection lasted up to 170 days. In a second experiment under higher fly pressure, diflubenzuron jetted at 2500 ppm protected sheep as long (about 56 days) as the standard, diazinon [144]. The efficacy of triflumuron against blowflies was reported in 1983. After topical application to gravid *L. cuprina* females, triflumuron completely inhibited egg hatching and was found to be more efficacious than diflubenzuron [184]. Similar results were obtained when gravid adult females received triflumuron mixed with the diet [185]. In a later study, the offspring of *L. sericata* adult flies exposed to targets impregnated with triflumuron was strongly reduced. Percentage egg hatch was about 3 to 10% compared with flies exposed to untreated targets [266].

### Commercial use

Commercial use of diflubenzuron and triflumuron against blowfly strike in sheep has been much more limited than against lice. Since its introduction, triflumuron (Zapp) was approved for use against flystrike in New Zealand but not in Australia [179]. Diflubenzuron (Fleececare) was initially approved against flystrike in both countries, but in 2008 claims for flystrike control were removed in all diflubenzuron products approved in Australia due to their inability to provide such control [8]. The exception is CLiK Plus, where diflubenzuron is combined with dicyclanil. No BPUs have been approved for use on sheep in Europe, where blowfly strike is a serious pest in Ireland, the Netherlands and the UK. High regulatory hurdles in the EU and/or commercial reasons may explain this absence.

## Tick control in cattle

Most BPUs used in animal health are highly effective against various insect species but show no efficacy against ticks at concentrations that allow their cost-effective use under practical conditions. In contrast with this, fluazuron is highly effective against immature stages of several tick and some mite species, but not against insects.

To better understand the usefulness of fluazuron for tick control in cattle, it is important to distinguish between one-host ticks, and two- or three-host ticks. Among the one-host ticks *R. (Boophilus) microplus* is the dominant species infesting cattle in many regions in tropical Australia, parts of Asia and Latin America, and its control represents the largest market for tickicides. *R. decoloratus* is found in numerous regions of East and Southern Africa. *R. annulatus* is another one-host tick occurring mainly in North America, parts of Europe and Asia, but of minor economic importance for the cattle industry. All these species are quite specific for cattle and wild bovids [121] and can occasionally infest horses and donkeys. *R. microplus* is the most investigated species regarding fluazuron efficacy.

Understanding the natural life cycle of these ticks is important for best use of fluazuron on cattle under field conditions. In one-host ticks, blood-engorged adult females detach from their host and drop to the ground. There they lay several thousand eggs and die. Larvae hatch out of eggs, find a host, attach, feed on blood and molt to nymphs on the same host. Nymphs remain attached to the same host, have their blood meal and molt to adults without leaving the host [221]. The parasitic life cycle of *R. microplus* on cattle lasts about three weeks and infestations are usually seasonal. Cattle infestation with larvae starts in spring, peaks in summer and recedes during the cold months of the year. The length of the tick season depends strongly on climatic conditions. During one season, 2–5 generations may follow. In most regions, cattle are virtually free of ticks during the cold season [221]. *R. annulatus* and *R. decoloratus* have comparable life cycles.

Multi-host ticks (two- or three-hosts ticks) are serious pests to cattle in subequatorial Africa, tropical parts of Latin America (e.g. in the Caribbean), and parts of Asia. Particularly important species are *Amblyomma hebraeum*, *A. variegatum* and *R. appendiculatus* in Africa, and *A. cajennense* in Latin America. The life cycle of three-host ticks differs from one-host ticks in that after their blood meal, engorged larvae and nymphs do not remain on the host but drop to the ground for molting as adults do for egg laying. This means that all three stages, larva, nymph and adult are free-living and can infest cattle (although for the larva and nymph, not exclusively), in contrast with one-host ticks, for which the only free-living infective stage is the larva [177].

### Efficacy

As for all BPUs, the basic effect of fluazuron on ticks is preventing molting from larvae to nymphs and from nymphs to adults by interfering with chitin synthesis. In addition, adult females treated with fluazuron produce normal amounts of eggs, but as it was demonstrated for fleas with other BPUs, no offspring will result because the newly developed larvae cannot hatch out of the egg envelopes [160]. Besides development disrupting effects, it has been shown that fluazuron also affects the salivary glands and the digestive system of ticks that had fed previously on treated cattle; such affected ticks showed abnormal shapes and colors and broke easily when detached by hand from the host [164]. A recent study has confirmed such effects of fluazuron on adult cattle ticks [120]. Investigations on nymphs of *R. sanguineus* indicate that fluazuron treatment damages many chitinous structures (smaller hypostome and chelicerae, scutum, sensilla, pores, anal plaque, etc.) that play essential roles for tick survival [34].

In laboratory assays, the IR50 (50% inhibition of reproduction) achieved by fluazuron against engorged adult *R. microplus* females (immersion test) was in the range of 3.5–12.5 ppm against various Australian strains and 26.5–47.0 ppm against various South American strains [160]. In trials *in vivo* on cattle experimentally infested with *R. microplus* larvae, the concentration of fluazuron in the blood of treated cattle needed to achieve 95% inhibition of reproduction was about 10 ppb against Australian strains, and 25–35 ppb against Latin American strains [160]. It was assumed that the difference between Australian and South American strains was related to the fact that Latin American *R. microplus* ticks are larger and about two times heavier than the Australian parasites. In the meantime, *R. microplus* from Australia and regions of Southeast Asia has been identified as *R. australis*, a separate species closely related morphologically but different from *R. microplus* [99].

If cattle are treated with fluazuron when they are already infested with one-host ticks, larvae will not molt to nymphs and nymphs will not molt to adults. Most adult females do not die but will complete their blood meal and drop after engorgement to lay eggs on the ground. After treatment with fluazuron, it usually takes about two weeks for infested cattle to become free of engorged adult ticks, although the offspring of most of these surviving ticks are not viable. From the perspective of most farmers, this means that the product does not kill the ticks. Cattle will carry engorged adult ticks for up to two weeks after treatment with fluazuron, much longer than after treatment with conventional adulticides.

However, if cattle are treated with fluazuron at the onset of the tick season (preventative treatment) before cattle become infested with the first larvae that become active in the pastures, these larvae will die after their blood meal. None or extremely few adult females will develop, complete engorgement, detach, lay eggs, and re-infect the pastures. Although cattle are actually infested with tick larvae, farmers will perceive their animals as tick-free because larvae are too small (<0.5 mm) to be noticed by the naked eye. Adult females are also the most damaging stage for cattle because each one ingests up to about 0.25 mL blood, which is much more than nymphs or larvae [221].

This preventative effect has been illustrated in studies carried out in Argentina [48]. In these studies, cattle treated with fluazuron at the recommended dose on day 0 were experimentally infested with *R. microplus* larvae three times a week until day 95 after treatment. The first engorged female tick that laid viable eggs was collected on day 63 after treatment. During the 95 days that the study lasted, percentage reduction of engorged viable female ticks collected was >99% compared with untreated cattle. The long residual effect achieved by fluazuron treatment means that in many cases, the entire tick season can be covered with 2–4 consecutive fluazuron administrations at intervals of 6–12 weeks, depending on cattle (type and breed), region and physiological status (i.e. lactating or not). Twenty-five years ago, when fluazuron was introduced, this was significantly longer than the 3–4 week treatment interval often recommended for obtaining a similar protection against one-host ticks [221, 275].

Such efficacy against *R. microplus* has been confirmed in field studies in several countries where these ticks are an important pest of cattle, e.g. in Brazil [4, 55, 196], Mexico [61], Australia [29], Argentina [214, 218, 219] and Colombia [16]. Field efficacy was also confirmed against *R. decoloratus* in South Africa [255]. All these studies showed that cattle treated at the recommended dose with a pour-on containing 2.5% of fluazuron did not develop infestations with adult ticks for periods between 8 and >12 weeks after treatment. Field studies also confirmed that efficacy against *R. microplus* depends on the type of cattle treated. Protection lasts longer in pure *B. indicus* breeds than in *B. indicus*–*B. taurus* crosses, or in pure *B. taurus* breeds. This is related to the known fact that *B. indicus* breeds have certain natural resistance to ticks, which substantially reduces survival of ticks on such breeds and their crosses with *B. taurus* [287]. In young cattle, rapid weight gains during the 8–12 weeks following treatment have the potential to reduce the length of protection provided by fluazuron treatment because it dilutes the fluazuron concentration in the blood and fat. However, this is rarely identified as being of practical concern in the field. Since fluazuron is partially excreted through the milk, protection of lactating cows is also shorter. However, since excretion in the milk is mostly in the form of the unchanged parent molecule, nursing calves that ingest fluazuron-containing milk do not need to be treated for protection against tick infestations, which represents a label claim of fluazuron products in several countries. In fact, fluazuron concentrations in plasma and fat of such nursing calves were found to be even higher than in their mother cows [94].

Interestingly, it has been shown that treatment with fluazuron does not hinder transmission of *Babesia bovis*, a major tick-borne disease and it does not interfere with the level of protective immunity of cattle conferred by tick infestations. Tick larvae can feed on blood on fluazuron-treated cattle long enough to ensure transmission of *Babesia* parasites at levels that support the natural build-up of natural immunity in treated cattle [142]. Recent studies have shown that fluazuron is also effective against *R. sanguineus*, a tick species not relevant for the cattle industry but an important parasite of dogs [34, 69–71].

In laboratory studies carried out during pre-clinical development, fluazuron was highly effective against numerous multi-host tick species of veterinary importance, including *A. hebraeum, A. variegatum, A. cajennense, R. appendiculatus*, *R. evertsi evertsi*, and *Ixodes ricinus* (Junquera, unpublished results). However, since fluazuron kills nymphs and larvae only when they attempt molting and it does not kill adult ticks, treating cattle with fluazuron will neither kill the ticks (larvae, nymphs or adults) already attached to the treated animals, nor prevent free-living stages of multi-host tick species from infesting the animals, feeding and engorging. Consequently, fluazuron has no curative effect but acts only as a long-term control of the tick populations. Where these ticks show seasonal development, strategic treatment of cattle herds at the onset of the tick season can significantly reduce cattle infestations by decimating the tick population in the pastures. This was shown in field trials in Mexico in 1994–1995 [195]. Two groups of 10 cross-bred cattle (Cebu-Brown Swiss) kept in separate paddocks were used for the trial. The first year, one group was treated twice (on May 14th and July 24th) at the recommended fluazuron dose of 2.5 mg/kg bw. The other group remained untreated. The second year, another group of 10 cattle was treated three times (on February 15th, May 11th and August 3rd) and the control group remained untreated until August 20th, when it had to be treated with an adulticide to reduce excessive ticks. *A. cajennense* nymphs and adult ticks were counted fortnightly in each group. Average reduction of the number of ticks during the whole treatment period of 24 weeks in 1994 was 70%. During the second year, reduction reached an average of 91% during the first 24 weeks, and dropped to 74% for the last 12 weeks following the lower number of ticks in the control group after adulticide treatment. From February 1997 to May 2000, a controlled efficacy study was conducted in the experimental farm of the South African Bureau of Standards in East London, South Africa [255]. Twenty-four Bonsmara cross bred steers (6/16 *B*. *taurus*, 5/8 *B. taurus africanus*) were divided into three groups of eight animals each. The three groups were allocated at random to three separate paddocks with similar vegetation and expected to contain similar numbers of *A. hebraeum*, *R. appendiculatus*, *R. evertsi evertsi* and *R. decoloratus*. Group 1 received no fluazuron treatment. Groups 2 and 3 were treated with fluazuron at a dose of 3 mg/kg bw every 12 or 8 weeks, respectively. On week 48 the animals were replaced. During the whole trial, animals of all groups were treated (spray-race) with amitraz when deemed necessary by the investigator. During the first 48 weeks, groups 1, 2, and 3 received 8, 9, and 8 full body tickicide treatments, respectively. The number of *A. hebraeum* and *R. decoloratus* counted on fluazuron-treated animals was substantially lower than in the control group, but not those of *R. evertsi evertsi* and *R. decoloratus*. Between weeks 48 and 168, groups 1, 2, and 3 received 23, 15, and 3 full body amitraz treatments. The different number of amitraz treatments between groups 2 and 3, both treated with fluazuron, was probably due to the fact that group 2 was treated every 12 weeks, and group 3 every eight weeks. The numbers of *A. hebraeum* and *R. appendiculatus* ticks in group 3 were significantly lower than in groups 1 and 2, and the tick numbers in group 2 were significantly lower than in group 1. No significant difference between the groups was found for *R. evertsi evertsi* ticks during this second period. The animals in groups 1, 2, and 3 gained an average of 159, 263 and 202 kg, respectively.

Published data regarding the efficacy of novaluron against *R. microplus* are inconsistent. In a field study, efficacy of a 5% novaluron injection administered at 5.0 mg/kg was compared with the original fluazuron 2.5% pour-on formulation (Acatak) administered at 2.5 mg/kg in cattle of a Nelore × Bonsmara cross naturally infested with *R. microplus* [272]. Efficacy was determined as the percentage reduction in the number of engorged female ticks collected between treatment and days 21–70 post-treatment in treated animals when compared with untreated ones. Average efficacy was 81.7% for novaluron and 84.4% for fluazuron. In another study, the efficacy of a 5% novaluron formulation administered topically as a pour-on at a dose of 2.5 and 5.0 mg/kg was investigated in Hereford/Charolais cattle (100% *B. taurus*) experimentally infested with *R. microplus* larvae [187]. The index of fecundity (IF) as described by Davey et al. [66] was determined daily for 27 days following treatment (therapeutic efficacy) and between days 28 and 48 after treatment (persistent efficacy) for both treatment groups. No significant reduction of the IF was found for either dose during the first 27 days after treatment (12.3 and 21.3% for the 2.5 and 5.0 mg/kg treatment, respectively). The level of control for the 2.5 mg/kg group was 69.5% at week 1 (day 34 post-treatment), 64.8% at week 2 (day 4 after treatment), and 28.0% at week 3 (day 48 after treatment), respectively. For the 5.0 mg/kg group, the level of control was 51.9% at week 1, 67.6% at week 2, and 34.1% at week 3. Both studies are not directly comparable and thus it cannot be excluded that novaluron is substantially more effective after injection than after pour-on treatment. The injectable formulation currently marketed in Brazil (Novatack Gold from Clarion) is a combination of 1% novaluron and 0.18% eprinomectin to be administered at a dose of 2 mg/kg novaluron and 0.36 mg/kg eprinomectin. A recent field study [192] in five different locations in Brazil investigated the efficacy of this commercial injectable formulation in mixed breed animals (*B. taurus* × *B. indicus*) naturally infested with *R. microplus*. Efficacy was determined as the percentage reduction in the number of engorged female ticks collected between treatment and up to 56 days post-treatment in treated cattle compared with untreated ones. Percentage control in all five locations did not exceed 48% any time after treatment. The study questions the suitability of this injectable formulation for the control of *R. microplus* on cattle when used as recommended by the manufacturer.

We found one study reporting efficacy of diflubenzuron against *R. microplus* larvae when administered to cattle mixed with mineral salts at an average rate of 30 mg/diflubenzuron daily during three months [5]. After repeated experimental infestations with *R. microplus* larvae, the number of engorged female ticks was counted every two weeks on both treated and untreated animals for one year, whereby treated and untreated animals bearing more than 100 engorged females were sprayed with a product containing 6% dichlorvos and 2% chlorpyrifos. After one year, the diflubenzuron-treated group showed a cumulative 57.6% reduction in the number of engorged ticks found, and whereas the diflubenzuron-treated group had to be sprayed 22 times with the acaricidal mixture, the untreated control group needed 76 spray treatments during the trial period. The authors also reported that the untreated group lost weight during the study (average of −12 kg/animal), whereas the treated group gained weight (+73 kg/animal). Based on histological studies, the authors concluded that diflubenzuron treatment had no effect on the fecundity of female adult ticks that completed engorgement, but acted to some extent only on the development of immature tick stages. These results are rather surprising, because so far no efficacy of diflubenzuron against cattle ticks has been reported, and diflubenzuron fed to cattle does not reach significant blood levels but is mostly excreted unchanged through the feces (see Section Diflubenzuron). Further studies would be needed to clarify and confirm or not this potential usage of diflubenzuron.

### Commercial use

Tick control on cattle has remained the only commercial use of fluazuron. This specificity – one target parasite in one target species – is quite exceptional for a veterinary parasiticide. This is also remarkable considering the projected high development costs of up to USD $50 m and the declining attractiveness of the tick control market, partially due to the discontinuation of large state-subsidized tick eradication campaigns in many countries such as Argentina, Kenya, Mexico, and Cuba [126].

After its introduction, usage of fluazuron was low to moderate due to the narrow spectrum of activity and its lack of killing effect on established tick infestations. Since then, use has steadily increased, driven by increasing resistance of one-host ticks *R. microplus* and *R. decoloratus* to many adulticides, first to synthetic pyrethroids and amitraz [100], and later to ivermectin [105, 197, 231] and fipronil [41, 42, 64, 206]. After expiry of patent protection, several companies have introduced their own fluazuron brands, containing either 2.5% fluazuron as the original pour-on brand, or various mixtures (e.g. with abamectin, cypermethrin, fipronil, flumethrin, ivermectin), for some of which efficacy studies have been published [40, 50, 51, 59, 110, 123, 189, 202]. An Internet search (October 2018) for commercial products containing fluazuron yielded 27 different brands in Australia, Latin America and South Africa from more than a dozen different companies, including most multinationals (e.g. Bayer, Elanco, Merial, Zoetis). The relative abundance of generic products indicates an increasing interest in fluazuron for tick control in countries where one-host ticks are a serious problem for the cattle industry.

Considering its recent introduction and so far only in Brazil, usage of novaluron against cattle ticks is probably still marginal. We did not find evidence that products containing diflubenzuron are being marketed for tick control in cattle in Brazil or elsewhere.

## Control of sea lice on farmed salmonids

Several marine copepodids are parasites to wild and farmed fish, with the salmon louse *Lepeophtheirus salmonis* (Copepoda: Caligidae) in the Northern Hemisphere and *Caligus rogercresseyi* (Copepoda: Caligidae) in Chile being the most economically important species [26, 54, 146].

The life cycle of *L. salmonis* proposed by Hamre et al. [131] progresses through eight instars after hatching, each separated by a molt of the exoskeleton to allow for growth and morphological development. The first two stages are free-living planktonic stages (nauplius I and nauplius II); the third stage, the copepodid is the infective stage that finds a host and attaches to its external surface; the following two stages (chalimus I and chalimus II) remain attached to the host by a short frontal filament that restricts their movement on the host; the three last stages (pre-adult I, pre-adult II and adult), are referred to as “mobile” stages since they move freely on the host’s surface and can move between hosts. *C. rogercresseyi* also progresses through eight instars although its life cycle differs from *L. salmonis* in that there are four attached chalimus stages and no mobile pre-adult stages [124]. *C. elongatus* and *C. clemensi* are also considered to be significant parasites in salmonid aquaculture in the northern hemisphere and in the north Atlantic and Pacific, respectively, although their infestations appear to be of less importance for farmed salmonids in respect to disease, compared to *L. salmonis* and *C. rogercresseyi* [155]. The economic impact of sea lice to salmonid farming worldwide have been estimated to about Euro 305 million [54], noting that these data are close to 10 years old and the sea lice situation has considerably worsened in the subsequent years.

### Efficacy

Teflubenzuron was reported to be effective against *L. salmonis* in 1996 [27]. The efficacy of a commercial in-feed formulation (Calicide^®^, Nutreco) was investigated in field studies in Norway and Scotland against natural *L. salmonis* infestations of farmed Atlantic salmon. Teflubenzuron was surface coated onto fish feed at a rate of 2 kg active ingredient per tonne of feed and administered to fish at a rate of 10 mg/kg bw/day for seven consecutive days. Maximum efficacy (83.4– 86.3% reduction) was reported against chalimus and pre-adult stages about two weeks after beginning the medication. As expected, the number of adult lice was not significantly reduced by the treatment, since they do not molt [27]. Comparable results were obtained in other field studies in Norway using the same formulation at the same dose rate against naturally infested farmed salmon at low water temperatures [245]. In these studies, a maximum reduction of up to 77.5% was achieved 26 days post-medication, whereby susceptible lice stages (chalimus and pre-adults) were reduced by a maximum of 88%. Similar efficacy was achieved in other field studies on farmed salmon in Canada carried out at the same in-feed dose rate as in the previously mentioned studies from Scotland and Norway [38, 39]. As can be expected for an IGD, these results show that teflubenzuron is effective against immature stages that have to molt to continue development, but not against adults that no longer need to molt. For practical purposes, this means that teflubenzuron is not adequate for rapid curative treatment of louse infestations, but rather for preventing their build-up. Under natural commercial conditions, season-dependent external recruitment of infective copepodids is usually unavoidable. Strategic treatments with teflubenzuron can prevent these copepodids from continuing development to more pathogenic pre-adult and adult stages and thus can keep the infestation below economic threshold levels. In contrast with the good efficacy obtained against *L. salmonis* on salmon, in a study in cultured sea bass (*Dicentrarchus labrax*) in Turkey, teflubenzuron-coated feed pellets administered to fish at a dose rate of 10 and 20 mg/kg bw for seven consecutive days had no effect on natural infestations with *Lernanthropus kroyeri*, a serious pathogen copepod of sea bass in the Mediterranean region. Whether the failure was due to insufficient dosing or other conditions (e.g. high water temperature) was not elucidated [278].

Diflubenzuron was also introduced as a lousicide on farmed salmonids in several countries, mainly in Chile and Norway [145, 191, 248], but we have not found original scientific reports on its efficacy against salmon lice in farmed salmon. Commercial products (e.g. Releeze vet from Ewos; CaliShot from FAV S.A.) are for oral administration, usually as medicated feed pellets administered at a standard dose rate of 3 mg/kg for 14 days [97]. Efficacy of diflubenzuron has been reported against other fish parasites of farmed fish. Fed to sea bass (*D. labrax*), diflubenzuron was found to be effective against the isopod parasite *Ceratothoa oestroides* in a study carried out in Greece, where this parasite is a frequent pest in farmed fish. Diflubenzuron was mixed with feed pellets and offered to fish kept in circular open tanks in order to achieve a dose rate of 3 mg/kg/day for 14 days. Nineteen days after treatment, no parasites were found on the treated fish [24]. Efficacy has also been reported against *Dolops carvalhoi*, a crustacean parasite of *Piaractus mesopotamicus*, the so-called “pez chato”, a ray finned fish endemic to the Paraná river that is also farmed in several Latin American countries. After administration of diflubenzuron incorporated into the feed for seven consecutive days at a dose rate of 0.935–1.291 mg/kg, 96.2–100% control was achieved [253].

More recently, lufenuron has shown efficacy against *L. salmonis*. In one investigation in Norway, the efficacy of lufenuron fed to smolt in fresh water hatcheries before transfer to sea was investigated [280]. Smolts were fed lufenuron medicated feed at 5 mg lufenuron/kg/day for seven consecutive days or a slightly reduced dose rate for additional days depending on their feeding behavior (total dose administered was 35 mg/kg). A control group was left untreated. The fish were transferred to marine cages after a short holding period. Such a treatment protected fish from being infected with *L. salmonis* for up to nine months. In a further study in Chile [186], smolts (mean weight 120 g) were fed lufenuron medicated feed shortly before sea transfer to allow dosing at a 0.5, 5 and 10 mg/kg/day for nine days to achieve total doses of 3.5, 35 and 70 mg/kg, respectively. Compared with untreated controls, the group that received the 3.5 mg/kg total dose was protected against *C. rogercresseyi* natural infestations for about 2.5 months, whereas the groups that received 35 mg/kg total dose or 70 mg/kg total dose showed a high level of protection for up to 6.6 months, compared with untreated controls.

In a recent investigation, first parasitic stages of *L. salmonis* exposed to 700 ppb lufenuron for three hours resulted in over 90% reduction in survival to the chalimus II life stage on the host, as compared to vehicle controls. Additionally, in a follow up *in vivo* administration study on the host >95% reduction of the chalimus I stage was observed. Transcriptomic responses of lice exposed to lufenuron included genes related to moulting, epithelial differentiation, solute transport, and general developmental processes. Global metabolite profiles also suggested that the membrane stability and fluidity was impacted in treated lice, possibly in vesicle transport. It was also observed that treated nauplii-staged lice exhibited multiple abnormalities in the integument, suggesting an impairment of the assembly of the epi- and procuticle [233].

### Commercial use

At the beginning of intensive aquaculture in Europe, the USA, Canada, Chile, etc., availability of parasiticides and other medications was very limited and dependent on the experience of carp culture from the Middle and Far East and from the ornamental fish trade [269]. In the 1970–1990s, several active ingredients were progressively introduced for louse control. Except for hydrogen peroxide, all had been previously used against pests of livestock or crops. They include organophosphates (azamethiphos, carbaryl, dichlorvos and trichlorfon), pyrethrum, synthetic pyrethroids (cypermethrin and deltamethrin) and macrocyclic lactones (emamectin benzoate, ivermectin) [127, 146, 248]. Teflubenzuron (Calicide) was introduced in the mid-1990s in several countries including Norway, Canada, Chile, Ireland and Scotland [78, 127, 248], but often under emergency conditions or with strong constraints regarding approved amounts and monitoring in order to minimize exposure to non-target arthropods in the marine environments [258]. More or less at the same time as teflubenzuron, diflubenzuron (Lepsidon) was introduced in Norway and Chile [248]. Both teflubenzuron and diflubenzuron were approved for in-feed administration, which is perceived by most producers as more convenient than topical (bath) treatment, the most common delivery method for other approved fish lousicides [127, 269]. Increasing resistance to organophosphates and synthetic pyrethroids favored the use of BPUs after their introduction about 20 years ago. However, the market share achieved by both compounds as well as the absolute amounts sold have been limited, e.g. a maximum of 1334 and 437 kg for teflubenzuron and diflubenzuron, respectively, in 1998 in Norway [78]. This was probably related to their lack of knock-down effect and their inefficacy against adult lice when compared with other compounds such as organophosphates, synthetic pyrethroids, hydrogen peroxide, or emamectin benzoate [127].

Later on, both compounds were almost abandoned and replaced by emamectin benzoate which is effective against both immature and adult stages and therefore appropriate for both prevention and rapid control of established infestations, and also approved for in-feed oral delivery [239]. However, after the appearance and spread of resistance to emamectin benzoate in numerous regions [98], both teflubenzuron and diflubenzuron have experienced a certain revival at the end of the last decade, e.g. in Norway and Chile [97, 145]. However, amounts used still remain low, e.g. in Norway of a total 3269 veterinary prescriptions associated with treatment of sea lice, only 201 prescriptions were reported for BPUs in 2015 [140]; and in Chile a maximum of 3878 kg diflubenzuron was used in 2009, compared with 10,524 kg of deltamethrin in 2008 [145]. BPUs are also increasingly being considered for use against fish parasites in Mediterranean aquaculture [10]

Approval of lufenuron (Imvixa, from Elanco) as a sea lice preventative was only recently granted in Chile and is still pending in other markets.

## Other studies on BPUs against veterinary parasites

Several BPUs have also been investigated against veterinary parasites other than those against which they have received regulatory approval. In the following section, we review the most relevant ones.

### Diflubenzuron

In an unusual investigation, diflubenzuron was incorporated into extruded pellets used for feeding rhinoceros in a wildlife park in Texas in order to achieve a dose rate of 1 or 0.1 mg/kg/day. Animals were administered medicated feed at the higher rate for 60 days and at the lower rate for 40 days, with an interval of 14 days without medication. Feces from treated animals were collected periodically and samples were seeded with eggs of laboratory-reared houseflies (*M. domestica*) and stable flies (*S. calcitrans*). Adult emergence was recorded in the samples. The content of diflubenzuron in the feces was also determined using liquid chromatography. At both dose rates, adult emergence in the feces was completely inhibited. Diflubenzuron content in the feces during the period of medication at 1.0 mg/kg/day ranged from 1.8 to 12.1 ppm. For the period of medication at 0.1 mg/kg/day, diflubenzuron residues in feces were below the limit of detection [291].

When incorporated into the larval rearing medium, diflubenzuron prevented adult emergence of the oriental rat flea, *Xenopsylla cheopis* [45] and of the cat flea, *C. felis* [91].

Several investigations have reported efficacy of diflubenzuron as a development inhibitor of tsetse flies. Topical treatment of gravid *Glossina morsitans morsitans* with diflubenzuron with as little as 0.5 μg/fly resulted in inhibition of viable offspring for more than 100 days after treatment [157]. Microinjection instead of topical administration achieved similar results [49]. In another study, 1 μg diflubenzuron topically applied to pregnant *G. palpalis palpalis* reduced viable offspring by 77–83% [162].

Diflubenzuron also showed efficacy as a feed-through against immature sand flies (*Phlebotomus papatasi*) after administration to Syrian hamsters (*Mesocricetus auratus*) mixed with the food for nine days in order to achieve average dose rates of 0.68, 6.26 and 66.28 mg/kg. Samples of the feces were periodically taken and bioassayed with laboratory-reared sand fly larvae. No larvae survived in the feces from animals that were fed diflubenzuron [198].

Applied to water ponds at a rate of 10–30 mg/L, diflubenzuron was efficacious against *Lernea cyprinacea*, a parasitic copepod infecting golden shiner minnows (*Notemigonus crysoleucas*), although parasites were not eliminated after a single treatment [30].

### Fluazuron

In an experimental study, dusky-wooded footrats (*Neotoma fuscipes*) were treated with fluazuron-containing baits to investigate whether it would control fleas (mainly *Orchopeas sexdentatus*) and ticks (*Ixodes* spp. and *Dermacentor* spp.) that transmit numerous human and veterinary diseases for which these footrats are an important host. Interestingly, after 3–4 months being fed with fluazuron-treated baits, the number of fleas was significantly reduced, but not the number of ticks [264]. The reasons for such unexpected efficacy of fluazuron against fleas would require additional investigations.

A clinical study to investigate the potential efficacy against natural infestations of *Demodex canis* of a fluazuron 2.5% formulation administered to dogs as a pour-on at a dose of 20 mg/kg, alone or together with ivermectin (0.6 mg/kg) concluded that neither fluazuron alone nor the combination with ivermectin were effective against canine demodicosis [271]. Fluazuron topically administered to pigs has been found to be moderately effective against *Sarcoptes scabiei* infestations [229].

In a series of studies conducted in South Africa, the effect of a commercial mixture of flumethrin and fluazuron for cattle (Drastic Deadline Extreme^®^, from Bayer) was investigated in sheep against blowfly larvae (*L. cuprina*) *in vitro* and *in vivo*, assuming that flumethrin would not have any efficacy against such larvae [11]. *In vitro* studies on blowfly larvae fed beef treated with the mixture at a concentration based on the registered dose of the product showed significant pupation defects in exposed larvae. Pharmacokinetic studies on treated adult sheep determined that virtually no fluazuron was absorbed trans-dermally into the bloodstream of treated animals but remained almost exclusively dissolved in wool fat or lanolin. Finally, blowfly larvae were exposed to sheep pelts treated or not with the mixture but no significant differences were found regarding macroscopic larval development and pupation, as well as hatching and adult fly development.

### Lufenuron

Lufenuron was not effective against generalized demodicosis (*Demodex* spp.) in dogs, even at dose rates substantially higher than those approved for flea control [256]. This is consistent with the low *in vitro* efficacy of lufenuron against ticks and mites found for lufenuron during pre-clinical development (Junquera, unpublished results).

Monthly oral administration of lufenuron at a dose rate of 10 mg/kg has been reported to be effective for the prevention of *Dermatobia hominis* and *Cochliomyia hominivorax* myiasis in dogs in Brazil [58].

Host-targeted feed cubes containing lufenuron fed to California ground squirrels (*Spermophilus beecheyi*) substantially reduced the population of fleas (*Oropsylla montana* and *Haplopsyllus anomalus*) after several single treatments per season [67]. In California, the ground squirrel and the two flea species mentioned are the main complex for amplifying epizootic plague by *Yersinia pestis*. A following study confirmed effective reduction of flea loads in Californian wild populations of ground squirrels, long-eared woodrats (*N. macrotis*), and mice (*Peromyscus* spp.) but not on Merriam’s chipmunks (*Tamias merriami*) after six years of systemic treatment via these feed cubes [68].

The effect of lufenuron topically administered to bed bugs (*Cimex lectularius* L) has also been investigated in laboratory studies. It was found that sub-lethal doses had a detrimental effect on the pulling forces of the legs that limit locomotion and could prevent bed bugs from moving within a domicile and taking a blood meal [35].

Lufenuron was also effective in controlling crustacean parasites (*Argulus* spp.) in frog tadpoles (*Rana hecksheri*) collected in a public park in South Carolina [289]. Lufenuron was found to be effective in controlling larvae of *Gnathia maxilaris*, a crustacean isopod of fish frequent in wild and captive fish populations. After oral administration to fish at a dose of 10 mg/kg bw once a month for six months, the parasite was eradicated from a large aquarium [139]. Efficacy of lufenuron dog tablets against fish lice (*Argulus* spp.) in Koi (*Cyprinus carpio*) has also been reported [200].

An unexpected effect of lufenuron has been reported on hydatid cysts (*Echinococcus granulosus*) when administered together with albendazole. In a study on mice, neither albendazole nor lufenuron alone showed an effect on the hydatid cysts. However, a single subcutaneous injection of lufenuron at high concentrations (45 or 100 mg/kg) combined with orally administered albendazole (50 mg/kg daily) caused ultrastructural alterations of the cysts’ walls and resulted in a 30–40% reduction of cyst growth. The mechanism responsible for such an effect was not investigated [28].

Abundant literature has also been generated regarding the antimycotic properties of lufenuron. The cell walls of fungi are known to contain chitin and the potential of lufenuron against fungal infections of dogs and cats was explored after it was introduced for flea control. Results are so far inconclusive. Whereas a number of clinical studies report successful control of various natural mycotic infections in dogs, cats, horses and chimpanzees, most studies under controlled *in vitro* or *in vivo* conditions have failed to confirm efficacy.

Antimycotic properties of lufenuron were first reported in 1997 after treatment of dogs suffering from pulmonary coccidioidomycosis caused by *Coccidioides immitis*. The animals were treated daily at a dose rate of 5–10 mg/kg for 16 weeks. All the dogs were reported symptom-free a year later [156]. Since the wall of *C. immitis* contains chitin, it was supposed that lufenuron also inhibited chitin synthesis in this organism. However, subsequent studies *in vitro* and *in vivo* on mice after oral and subcutaneous administration failed to confirm this efficacy [133, 156]. Lufenuron was also found to be active *in vitro* against *Encephalitozoon intestinalis* and *Vittaforma corneae*, two agents of human and animal microsporidiosis [81].

In a clinical study, 129 dogs and 159 cats naturally infected with dermatophytes were treated once orally at 54.2–68.3 mg/kg (dogs) or 51.2–266 mg/kg (cats). Dermatophytosis persisted for 2–3 months in untreated animals, whereas animals treated with lufenuron recovered much faster: cats within 10–15 days and dogs within 16–25 days. Analysis of infections identified *Microsporum canis* and *Trichosporon mentagrophytes* in dogs and cats. In dogs *M. gypseum* was also found, as well as a few cases of superficial dermatomycosis by *Aspergillus niger* and *Candida albicans* [15]. In another study, cats naturally infected with *M. canis* were treated four times orally with lufenuron at 120 mg/kg every three weeks. All but one of the animals recovered within 21–42 days after treatment initiation [237]. A slightly lower success rate was reported in another study on dogs and cats naturally infected with *M. canis* and *M. rubrum* [270]. Efficacy against *M. canis* in cats was also reported for lufenuron at 60 mg/kg administered orally on day 0 and 30, in combination with weekly enilconazole rinses [128]. A high curative efficacy (98%) 45 days after the last treatment was again reported on cats naturally infected with *M. canis* treated orally with lufenuron at 120 mg/kg four times at a 3-week interval [238].

Lufenuron was also reported to effectively cure various natural cutaneous mycosis on five chimpanzees (*Pan troglodytes*) held in a wildlife center for orphaned or injured wild animals in Cameroon. Diagnosis before and after treatment was done after visual examination because skin scrapings would have required anesthetizing the animals, which was rejected for animal welfare reasons. Two oral administrations of lufenuron (mixed with the feed at a dose of 60 mg/kg) with a three-week treatment interval were sufficient to cure the infections. One animal that had a four year-long history of repeated recurrent infections after apparently successful treatments with various antimycotic medicines (e.g. ketoconazole, griseofulvin, miconazole) received a third lufenuron treatment 10 weeks after the first one. In this particular chimpanzee, several previous laboratory diagnoses had confirmed *Aspergillus* spp., *Candida* spp. and *M. canis*. In this chimpanzee, and three other animals, clinical signs strongly improved three weeks after the first lufenuron treatment. In one animal, it took seven weeks for the improvement to become evident. All five chimpanzees remained healthy 6–8 months after the last lufenuron treatment [87].

However, lufenuron administered orally at the dose recommended for flea control (i.e. between about 30 and 133 mg/kg) did not prevent cats from becoming infected with dermatophytosis after direct topical challenge with *M. canis* [216] or after contact with other infected cats [75]. Similar negative results were reported after *in vitro* and *in vivo* studies against *M. canis*, *M. gypseum* and *T. mentagrophytes* in dogs and cats [294] and in a guinea pig model [282]. In one study, lufenuron showed neither an individual nor a synergistic effect when administered together with terbinafine to cats experimentally infected with *M. canis* [76]. In another investigation involving 100 cats in two catteries, animals naturally infected with *M. canis* treated with both enilconazole (weekly rinses during four weeks) and lufenuron (oral treatment at 60 mg/kg at days 0 and 30) showed significantly lower clinical scores 30 and 60 days after treatment initiation than cats treated with enilconazole (as previously described) and micronized griseofulvin (25 mg/kg) administered orally twice a day for five weeks) but not with lufenuron. In both treatment groups, the mean number of fungal colonies increased from day 60 after treatment until the end of the study on day 90 [128]. In another study involving 50 cats naturally infected with *M. canis*, oral lufenuron treatment at 100 mg/kg every 15 days during 60 days followed by enilconazole or griseofulvin treatment was more effective than without pre-treatment, whereby lufenuron pre-treatment alone was not effective at all. The authors propose an immunomodulatory effect of lufenuron, and suggest that it may be particularly useful against long-lasting infections, unsuccessfully treated with conventional drugs [193]. In a recent review, lufenuron has not been recommended for the treatment of dermatophytosis in cats due to inconsistent results [118].

Potential use of lufenuron as an antimycotic in horses has also been explored and off-label use of lufenuron by veterinarians is reported against various fungal infections in horses, e.g. endometritis, guttural pouch mycosis, paranasal sinusitis and keratomycosis [257]. One study in four mares reported effective control of fungal endometritis after intrauterine lavage with lufenuron suspended in sterile saline solution [134]. These infections are known to be associated with equine infertility. However, another study on horses failed to confirm efficacy *in vitro* against *Aspergillus* spp. and *Fusarium* spp. isolated from infected equine cornea and concluded that the concentrations of lufenuron in blood of horses treated orally at various dose rates recommended among veterinarians was lower than those proven to be ineffective *in vitro* [257].

Use of lufenuron against human fungal infections, particularly those caused by *C. albicans* has become a major topic in social media. Google search results on the term “lufenuron” serve numerous links to pages dealing with off-label uses in humans. To our knowledge, no scientific investigations have been published so far on this topic.

### Novaluron

We have not found reports in the literature regarding studies with novaluron other than those previously cited [187, 188, 192, 272].

### Teflubenzuron

In a laboratory study on Nile tilapia (*Oreochromis niloticus*) and pacu (*Piaractus mesopotamicus*), the efficacy of teflubenzuron against *Trichodina* spp., a protozoan parasite, was investigated under laboratory conditions [147]. Treatment consisted in a daily therapeutic bath for five consecutive days with an interval of 24 h. Tilapia were bathed at concentrations of 30 or 50 mg teflubenzuron/L for 1 h, and pacu at concentrations of 30, 50 and 80 mg teflubenzuron/L for 2 h. In tilapia treated at 50 mg/L, an average reduction of 87.9% in the number of parasites was achieved. In pacu, the highest reduction of 96.1% was obtained after the 80 mg/L treatment.

### Triflumuron

In laboratory tests, triflumuron inhibited the production of viable offspring of tsetse flies (*G. morsitans morsitans*) topically treated or exposed to tarsal contact with impregnated targets. Such targets retained their efficacy after six months’ exposure to field conditions [178]. Triflumuron was also efficacious against larvae of the cat flea (*C. felis*) when admixed to the larval rearing medium (LC50 of 0.36 ppm) [91].

Triflumuron showed unexpected efficacy against free-living stages of sheep nematodes. Efficacy was high against larvae of *Trichostrongylus colubriformis*, but lower against related nematodes such as *Haemonchus contortus* and *Ostertagia circumcincta* [285]. To our knowledge, no such nematocidal effect on roundworms has been reported for other BPUs.

## Fate of BPUs in treated domestic animals and fish

The usefulness of any medication depends not only on the intrinsic properties of its active ingredient, but also on its fate once administered. In the context of ectoparasite control on domestic animals, a key question is whether an active ingredient has a systemic effect or not, i.e. whether or not it is absorbed by the host and reaches the parasite through the blood or the tissues of the host. As described in detail later in this section, it can be concluded that in approved uses on mammals, lufenuron (dogs and cats) and fluazuron (cattle) show a systemic mode of action. After topical administration to cattle, novaluron also has a systemic mode of action, but it has not been investigated whether this is also the case after topical administration to dogs. When fed to salmon or salmonids, diflubenzuron, lufenuron and teflubenzuron show a systemic mode of action as well. In contrast with this, diflubenzuron in cattle and sheep, and triflumuron in sheep do not show a systemic mode of action.

### Diflubenzuron

After topical administration to cattle in the form of a wettable powder, diflubenzuron was not absorbed through the skin to any significant extent [227]. After oral administration of radio-labeled diflubenzuron, about 85% of the administered dose was eliminated in the feces, about 15% in the urine and about 0.2% was secreted into the milk. Unmetabolized diflubenzuron was the major component in feces, where seven metabolites amounted for about 20–25% of the excreted radioactivity [149]. Almost no detectable levels were found in blood or fat after oral administration. Residues in milk of dairy cows were low, consisted mainly in various metabolites, and were undetectable after 3–4 days [227]. In a recent study, *B. indicus* bulls and Girolando dairy cows (3/8 *B. indicus* Gyr × 5/8 *B. taurus* Holstein) received diflubenzuron mixed to the feed in order to achieve a dose of 30 mg/kg/day for 120 days (bulls) and 77 days (cows). Tissues and milk samples were analyzed for residues 0, 12 and 24 h after interrupting treatment with diflubenzuron. In all samples analyzed, diflubenzuron residues were below the detection limit of <0.006 mg/kg for fat and 0.0006 mg/kg for milk [277].

In sheep treated orally with diflubenzuron, 42 and 41% of the administered dose was eliminated through feces and urine, respectively. Low residues were found in the liver and the kidneys. Residues in other tissues were below the detection limit [227]. After topical administration (hand-jetting) to long wool sheep, diflubenzuron had a very slow breakdown rate, starting with a half-life of 178 days and averaging 238–284 days over the next 3–5 months [37]. When applied by dipping or hand jetting, the average residues in wool 12 months after treatment were 20–40 mg/kg [215].

In goats treated orally with diflubenzuron, residues were eliminated mainly in the feces, only 3.9–14% was eliminated through urine. Only about 0.1% of the administered dose was found in milk, mainly in the form of various metabolites, with a peak between 8 and 24 h after administration. Residues in fat and muscle were below the detection limit [227].

In pigs, after oral administration, 82% of the dose was recovered in the feces as unchanged diflubenzuron, and 5% in the urine in the form of various metabolites. Residues in tissues were rather low (<0.5 mg/kg), particularly in subcutaneous fat (0.2 mg/kg) and blood (0.06 mg/kg) [224].

The fate of diflubenzuron in laying hens was investigated in two different breeds, White Leghorns (WL) and Rhode Island Red/Barred Plymouth Rock Buff (RIR). Eight hours after administration, almost half of the dose was already recovered in the excreta. Altogether 82% (RIR) to 91% (WL) was recovered from the excreta, out of this only 3.4% (RIR) to 16% (WL) were metabolites, and the rest was unchanged diflubenzuron. Residues in eggs ranged from 0.16 (RIR) to 0.25 (WL) mg/kg 3–6 days after treatment. Residues in body fat were very low, 0.007 mg/kg (WL) to 0.043 mg/kg (RIR) [223, 225]. In an already mentioned study [210] where hens received medicated feed containing 10 ppm diflubenzuron for 15 weeks, residues in eggs reached 0.38–0.55 ppm.

These data show that after oral administration, diflubenzuron is poorly absorbed in the gut of livestock and poultry and most of it is excreted in the feces, mainly as the unchanged parent molecule. This allows the build-up of concentrations in the dung that are effective against dung-breeding flies, hence it is adequate for feed-through control of these parasites. In contrast with this, absorbed diflubenzuron is quickly metabolized, mainly in the liver, and the various metabolites are quickly excreted through feces and urine. As a consequence, diflubenzuron levels in blood remain very low and are unlikely to exert any therapeutic effect on blood-sucking flies such as horn flies or stable flies, or on other hematophagous ectoparasites (e.g. fleas, tsetse flies, etc.) that otherwise would be susceptible to diflubenzuron. After topical administration, diflubenzuron remains mainly in the hair coat of treated animals for months. Very little is absorbed through the skin and what is absorbed (probably also through licking) is quickly metabolized and excreted. In sheep, lice or blowfly maggots are affected by contact or ingestion of diflubenzuron in the wool, not from the tissues of the host. In other words, diflubenzuron has basically a non-systemic mode of action on livestock and poultry.

In Atlantic salmon (*Salmo salar*), after oral administration of diflubenzuron at high doses (75 mg/kg, i.e. 25 times the recommended dose against *L-salmonis*), only 3.7% was absorbed after 12 h. After treatment at the recommended dose, bioavailability was calculated to be 31% at a water temperature of 6 °C, but is temperature and dose dependent, and saturable, i.e. a higher intake does not necessarily result in higher absorption. Mean plasma levels (0.141 μg/L) were reached after 24 h. Highest residues were found in the liver, but they accounted only for 0.3% of the administered dose. Elimination half-time was calculated to be 71.4 h [92]. Residues in fillet and skin tissues were also low, but higher than the minimum effective concentration (MEC) of 900 μg/kg assumed by the industry to be effective against sea lice [97].

In Atlantic cod (*Gadus morhua*), gastrointestinal absorption of orally administered diflubenzuron is probably lower than in Atlantic salmon. In a pharmacokinetic study, residues of diflubenzuron found in cod tissues (fillet and skin) were only about 1.5% of those found in Atlantic salmon after similar dosing, and the author suggests that this will probably make diflubenzuron unsuitable for the control of louse infestations in this species [97].

It is generally assumed that diflubenzuron administered orally to salmon acts systemically on parasitic sea lice, i.e. through the tissues of the host, where residues reach concentrations effective to inhibit development of immature louse stages that feed on it and are exposed to fish mucus [102].

### Fluazuron

Besides macrocyclic lactones, fluazuron is currently the only active substance that has a systemic mode of action and is commercially used to control cattle ticks. Being highly lipophilic, fluazuron adheres to the lipids in the hair coat of the host. From there, it is slowly absorbed into the blood, partly through the skin, partly through licking, but the ratio for percutaneous vs. oral absorption has not been determined [94, 234]. In cattle, absorption through licking has been shown to play an essential role after pour-on administration of ivermectin, another parasiticide with a systemic mode of action [25, 175, 176]. It was shown that self- and allo-licking may account for 58–87% of the total ivermectin intake, compared with only 10% absorbed percutaneously [176]. In the case of fluazuron, oral intake through licking does not negatively influence efficacy, it may even be favorable since absorption to blood is even faster and results in higher blood levels after oral than after dermal treatment [276]. After topical administration to cattle at the dose recommended in Australia (1.5 mg/kg), the total intake of the administered dose was at least 60% [234]. Maximum plasma levels were observed 48 h after administration and a steady state between absorption and elimination was observed between three and four weeks after treatment [94, 234]. Body fat is the preferential tissue for fluazuron, where it is found at about 10–20 times higher levels than elsewhere [276]. Highest residues were found about two weeks after treatment in renal fat (4.8 mg/kg fluazuron equivalents), omental fat (4.3 mg/kg), subcutaneous fat (ventral: 3.9 mg/kg; dorsal: 2.8 mg/kg), and skin (3 mg/kg). Lower levels were found in the liver (0.5 mg/kg), kidney (0.4 mg/kg), muscle (0.1 mg/kg) and brain (0.08 mg/kg). The depletion half-time for the different tissues varied from 4.5 to 5.5 weeks, but was 1.5 weeks for skin. After pour-on treatment at 1.5 mg/kg, the mean plasma levels (those relevant for tick control) remained quite stable between 9 and 35 days after treatment, ranging from 35 to 41 ppb and declined thereafter with an elimination half-life of 73 days. Sixteen weeks after treatment, plasma levels were about 7 ppb [234].

Fluazuron is poorly metabolized, mainly in the liver. The parent molecule accounted for more than 90% of the residues in tissues and feces [94]. The major excretion route was the feces (40–62%) and only 1% was eliminated through urine [234].

Altogether, the fate of fluazuron on cattle after topical administration can be described as a two-compartment model with a prolonged infusion phase from the skin depot. During the distribution phase, equilibrium between fat tissues and plasma is established and maintained for several weeks during the excretion phase. This results in a natural slow-release mechanism that ensures persistent bioavailability of fluazuron for tick control for a number of weeks.

### Lufenuron

After ingestion by dogs, only about 40% of the administered lufenuron is absorbed in about 6 h, and about 60% is excreted unchanged in the feces [6]. A maximum blood level is achieved about 8 h after administration [89]. In dogs, after absorption to the blood, it is distributed to body tissues and is stored preferentially in body fat, from which it is released back to the blood with a half-life in fat between 15 and 50 days [283]. Excretion is very slow and predominantly in feces via a non-biliary process (33% within 24 h, still measurable after 21 days) mainly as the unchanged parent compound with only about 1% being metabolized. Bioavailability of absorbed lufenuron was estimated to be 70% [89]. The fate of lufenuron in the host’s body represents a two-compartment model where equilibrium between fat tissues and plasma is maintained, thereby keeping the blood concentration of lufenuron at levels that ensure complete inhibition of hatching of eggs produced by the adult female fleas for several weeks [293]. Monthly oral re-treatments ensure that the effective blood concentration of lufenuron is maintained across the whole flea season. This is usually sufficient to completely eliminate the flea population from a household, provided that all dogs and cats in the same household are treated and there is no external source of flea infestation.

After treatment of cats with the injectable instead of the oral formulation, a similar blood-fat equilibrium is established that ensures effective blood levels of lufenuron for up to six months after a single treatment. In a pharmacokinetic study, after injecting lufenuron in cats at a dose of 5 or 10 mg/kg, peak plasma levels were achieved 8.2 weeks after treatment at 194.3 ng/mL and 388.7 ng/mL, respectively. Plasma levels remained above the MEC of >100 ng/mL for 26 weeks after treatment at 10 mg/kg [116]. Interestingly, a single oral treatment of cats at a dose of 30 mg/kg ensures about 1 months’ efficacy, whereas a single injection at a dose of 10 mg/kg ensures about six months efficacy [254]. This is likely to be related to the poor absorption of lufenuron after oral administration, since the bioavailability of absorbed lufenuron was found to be similar (about 70%) following oral or parenteral (intravenous) administration to mammals [89]. In a specific study in dogs, it was shown that after oral treatment at the recommended dose, no lufenuron residues were found on the skin surface of treated animals [143]. This is considered a safety advantage when compared with topical products, e.g. for spot-on application.

In fish, metabolism studies on the fate of lufenuron have been reported in the literature in bluegill sunfish and fathead minnow but not in salmonids. In bluegill sunfish, the only residue present was the parent compound and in fathead minnow, 91–96% of the residues were characterized as lufenuron [96]. Although the data are not publicly available, a radiolabeled ADME study with [^14^C]-lufenuron in Atlantic salmon was evaluated by the US FDA to establish an import tolerance for lufenuron in Atlantic salmon. It was concluded that lufenuron was not extensively metabolized in Atlantic salmon and the parent compound was the major component of the total residues in fish muscle and skin [104]. The studies conducted in bluegill sunfish and fathead minnow did not reveal significant differences in the kinetics and metabolism of the substance compared to mammals and birds [96], which allows us to conclude that lufenuron fed to fish for the prevention of sea lice also has a systemic mode of action.

### Novaluron

The fate of novaluron has been investigated in lactating goats and hens after oral administration of radiolabelled material [101]. Novaluron was poorly metabolized in both species. Most of the radioactivity (52–72%) was excreted through the feces. In goats, the highest residues were found in peritoneal fat and milk, in hens in body fat and eggs. In a study in cattle in Brazil [272], the concentration of novaluron in blood plasma after a single topical treatment (pour-on) at a dose of 2.5 or 5.0 mg/kg resulted in maximum levels of 378 and 396 ng/mL, respectively, and remained above the threshold concentration of 100 ng/mL until days 28 and 42 after treatment, respectively. These data suggest that novaluron topically administered to cattle is progressively absorbed to blood during the first days after treatment from where it is ingested by infesting ticks and tick larvae, similar to what has been reported for fluazuron in cattle. Whether and where novaluron is stored (body fat, hide, etc.) was not investigated. We have not found studies on the fate of novaluron after topical administration to dogs.

### Teflubenzuron

The fate of teflubenzuron in salmon after oral administration has been investigated in several studies [245] and is summarized in the Summary Report of the European Agency for the Evaluation of Medicinal Products issued for the approval of the maximum residue limit (MRL) for teflubenzuron in salmon [93]. After a single oral administration of 10 mg/kg, the highest mean plasma concentration was obtained 9–24 h post-dose, depending on water temperature. The plasma levels decreased subsequently with half-lives of 15–20 h. After repeated administration of 10 mg/kg bw/day for seven consecutive days, steady-state levels in plasma were achieved after about three days, and the elimination half-life was about 23 h. Highest amounts were found in the gall bladder, liver and kidneys [93]. Metabolites found in fish are the same as those found in rats. However, teflubenzuron is poorly metabolized in salmon. In muscle and skin, only unchanged teflubenzuron was found on days 1 and 8 after single dosing. In the liver and kidney, unchanged teflubenzuron was the major component, 77 and 69%, respectively at day 1 after treatment. Globally, the bioavailability of orally administered teflubenzuron was low, 4–9% at 9 °C and 14 °C, respectively, indicating temperature-dependent absorption [93]. On average, only about 10% of the administered teflubenzuron is absorbed from the gastrointestinal tract, i.e. about 90% is excreted in the feces, mainly as the unchanged parent molecule [258]. It can be assumed that teflubenzuron orally administered to salmon acts systemically against sea lice, i.e. it is absorbed into the host’s blood and distributed throughout its body. Lice feeding on the host’s tissues and ingesting enough teflubenzuron are likely affected when feeding and/or exposed to fish mucus.

### Triflumuron

Data on the fate of triflumuron in the host after topical administration to sheep, cattle or horses could not be found in the literature. Based on safety data for a crop protection formulation, it can be assumed that it is poorly absorbed through the host’s skin or through licking and that it acts directly on lice or blowfly maggots, either through contact or through ingestion of the active ingredient deposited on the host’s hairs or skin, and not through the host’s blood. Based on the usual toxicological studies (rat, rabbit, etc.), a low dermal absorption of between 1 and 5% has been reported for a crop protection formulation [90]. The same document indicates that after oral administration, 78– 96% of the administered dose is absorbed into blood and the maximum concentration in most organs was reached one day after administration, up to three days in blood. However, the amounts of residues found were low and rapid excretion was observed, 89–95% within 48 h via urine and feces. The low absorption rate after topical administration and the rapid excretion suggest that the concentration of triflumuron in the host’s blood after a single pour-on administration would not be high enough to ensure systemic efficacy over several months. It has also been shown that after off-shears (i.e. within 24 h of shearing) topical administration, significant amounts of triflumuron (average 30 mg/kg wool) remain in the fleece of sheep for up to 12 months [215]. In another study on triflumuron persistence and distribution in sheep fleece, it was found that 12 months after off-shears pour-on treatment most of the active ingredient was found on the tip of the fleece (43.9–73.5%), and very little (<1%) on the base. This very low mobility of triflumuron in the fleece is consistent with the fact that this compound is a crystalline solid that is poorly soluble in water and lipids, and consequently it is unlikely that it is washed down to skin level by rain or that it diffuses in wool wax in the fleece. To explain the proven high efficacy of triflumuron against sheep body lice, the authors suggest that either the active agent is potent enough at the low concentration found at the skin level, or lice migrate in fleece to the wool “canopy” where they are exposed to higher concentrations [250]. Altogether, triflumuron was found to be quite persistent in sheep wool. In another study where triflumuron was applied off-shears as a pour-on, the initial half-life was 95 days, increasing to 122 days after 12 months, and averaging 119 days over the year [37].

In a study on mini-broiler breeder hens that were treated with triflumuron added to the feed at 15–25 ppm for four weeks, triflumuron residues were found in the eggs from hens treated at concentrations of >5 ppm in the feed [213].

## Drug resistance

Reviewing resistance of pests to BPUs a few years ago, Doucet and Retnakaran [83] indicated that resistance was reported for all but four of the BPUs that had been marketed so far: fluazuron, hexaflumuron, noviflumuron and novaluron. However, resistance to hexaflumuron has been reported in Egyptian field strains of the cotton leafworm, *Spodotera littoralis*, with a resistance ratio (RR, also called Resistance Factor) between 217 and 533 [9] and reduced susceptibility (2.5-fold) to novaluron has also been reported in field strains of the Colorado potato beetle, *Leptinotarsa decemlineata* [65].

Resistance of veterinary parasites to BPUs has been poorly analyzed so far. In a review study from 2017 [273], no single case of veterinary parasites resistant to these compounds is mentioned, although resistance of houseflies, blowflies and sheep lice to diflubenzuron and of sheep lice to triflumuron had already been reported earlier. In the meantime, as described below in detail, resistance of cattle ticks (*R. microplus*) to fluazuron has already been reported. Out of the six BPUs used as veterinary parasiticides, resistance has been confirmed in three of the major target parasites (blowflies to diflubenzuron, sheep body lice to diflubenzuron and triflumuron, and cattle ticks to fluazuron). We have reviewed the literature on resistance focusing on the time it has taken to develop in a particular usage and, if reported, how strong it has become as indicated by the RR, i.e. the ratio between the concentrations needed to kill resistant and susceptible parasites.

### Diflubenzuron

Already in 1974 Cerf and Georghiou [43] reported cross-resistance of diflubenzuron in several housefly strains that had been reared and selected in the laboratory for organophosphate, organochlorine and carbamate resistance and had never been exposed to diflubenzuron, with RRs of 10 and higher. Similar findings with laboratory-selected housefly strains were reported in 1977 showing RRs of about 50 [226]. In further investigations, a diflubenzuron-resistant housefly strain was selected in the laboratory that reached an RR of >1000. The authors also investigated the resistance mechanisms involved and concluded that it was due to the combined effect of reduced cuticular penetration, increased metabolism due mainly to mixed function oxidase enzymes, and rapid excretion of the chemical [232]. Since then, resistance of arthropod pests to diflubenzuron and other BPUs as well as side-resistance among BPUs has been widely investigated and reviewed several times (e.g. [83, 148, 230]).

Among veterinary parasites, cross-resistance to diflubenzuron was reported in the larvae of organophosphate-resistant strains of the Australian sheep blowfly (*L. cuprina*) before it was introduced for use on sheep in Australia, with RR > 1000 in laboratory-selected strains and evidence for enhanced detoxification through increased monooxygenase activity in the resistant strains [166, 167], a detoxification mechanism also found in organophosphate-resistant blowflies [166]. Subsequent studies suggested that other mechanisms than monooxygenases could be involved as well [168]. High levels of resistance were later reported for field strains of *L. cuprina* collected after reported product failures, with one strain exhibiting a 790-fold resistance to diflubenzuron [181, 183]. Tolerance of *L. cuprina* to diflubenzuron was also confirmed in New Zealand [129]. Resistance to diflubenzuron has also been reported in Australia for the sheep body louse (*B. ovis)*. First reports were confirmed in laboratory assays conducted on several field strains collected in sheep farms and showed RRs ranging from 2.4 to 90.1 and clear side-resistance with triflumuron [153, 182]. Considering that diflubenzuron was introduced in Australia in 1993 [141], it took about 12 years for louse resistance to be reported. So far, louse resistance to diflubenzuron seems not to be present in New Zealand [132].

When diflubenzuron was introduced for use on sheep in Australia, blowfly resistance to organophosphates was already high and widespread [179]. Since diflubenzuron shows high cross-resistance to organophosphates, field resistance to diflubenzuron was already there and needed only a few years to be confirmed. In contrast with this, organophosphate resistance to lice was low and rare in Australia when diflubenzuron and triflumuron were introduced. The reason is probably that synthetic pyrethroids had dominated the lousicide market until then, and they do not show cross-resistance with BPUs. Synthetic pyrethroids had largely displaced organophosphates as lousicides before high levels of resistance had developed. Their success was based on the lower toxicity of synthetic pyrethroids and the ease of use as ready-to-use backliners. Lice resistance to synthetic pyrethroids developed rather quickly. However, although for some strains it could be shown that synthetic pyrethroid resistance was suppressible by co-treatment with monooxygenase inhibitor piperonyl butoxide, no known synthetic pyrethroid-resistant lice populations were found that were not susceptible to organophosphates [179]. This probably explains why BPUs did not encounter cross-resistance with synthetic pyrethroids when they were introduced. When resistance to synthetic pyrethroids made them unacceptable for louse control, they were quickly substituted by BPUs [179]. This probably explains why field resistance to BPUs took substantially longer to develop in lice than in blowflies.

Field resistance of houseflies to diflubenzuron has been reported in numerous countries, but mostly at low to moderate levels (i.e. RRs of <10), e.g. in Denmark [163, 170], Turkey [44] and Hungary [228]. We have not found reports confirming resistance of horn flies, face flies or stable flies to diflubenzuron or other BPUs. This is probably related to the very scarce use of these compounds for the control of dung-breeding flies that parasitize cattle.

Tolerance (i.e. low resistance with RR of 3–5) or resistance of sea lice to diflubenzuron has not been reported yet [1], which is not surprising considering its rather limited use so far.

### Fluazuron

*R. microplus* is the tick species with the most severe resistance problems worldwide. It has successively developed resistance to all available chemical classes of acaricides used for its control during the last century [100, 122]. Resistance of *R. microplus* to fluazuron was first reported in Australia in 2010 in ticks collected from three properties in Queensland. Laboratory assays concluded an RR of about 20. Data were presented in a congress poster [150]. Whether these ticks resistant to fluazuron were also resistant to other acaricides was not reported. However, we have not found published studies confirming and/or characterizing this reported case or reporting on newer cases in Australia since then. An RR of 20 does not appear to be alarming, considering that RRs of over 1000 are not uncommon for resistant strains of numerous arthropods, both for some adulticides and for BPUs. However, in the case of fluazuron, the levels of active ingredient to which the parasites are exposed on treated cattle are usually not higher than four-fold the minimum effective concentration (MEC), in contrast with other acaricides, particularly those used for dipping and spraying, for which the treatment concentration may be 50-fold and higher than their MEC against ticks (e.g. with synthetic pyrethroids). As previously described, the concentration of fluazuron in blood to achieve 95% inhibition of reproduction (IR95) of Australian *R. microplus* strains is about 10 ppb; and topical treatment at 1.5 mg/kg as recommended in Australia ensures maximum plasma levels of 35–41 ppb, i.e. about four times higher than the IR95. It is obvious that an RR factor of 20 is sufficient to dramatically reduce the efficacy of topically administered fluazuron.

Reck and colleagues [241] reported in 2014 the first confirmed case of *R. microplus* resistance to fluazuron in Latin America. It was detected in the “Jaguar” strain collected from a property in Southern Brazil (Rio Grande do Sul). This property had a history of very intensive use of acaricides, alternating a chlorpyrifos/cypermethrin mixture (pour-on or plunge dipping) with fluazuron (pour-on) and long-acting avermectins (injectables) during the last years. This strain has been shown to be also resistant to active ingredients of five other chemical classes: organophosphates (chlorpyrifos), synthetic pyrethroids (cypermethrin), amidines (amitraz), phenylpyrazoles (fipronil) and avermectins (ivermectin). In a field trial, two groups of 20 cattle each were artificially infested with larvae from the Jaguar strain or from a susceptible strain and treated with fluazuron at the recommended dose. Between 14 and 28 days after treatment, the average efficacy in cattle infested with the susceptible strain was 96%, while for the Jaguar strain the efficacy was zero. In a modified artificial immersion test, 50 ppm of fluazuron inhibited 99% of larvae hatching in the susceptible strain and less than 50% in the Jaguar strain. A separate investigation on ticks of this strain found a significant elevation in MFO contents and esterases activity in the resistant strain when compared with the susceptible strain, in eggs and larvae, respectively [119]. MFOs are known to be involved in the detoxification of numerous other chemical classes and their higher content in this strain probably explains its resistance to so many chemical classes.

Fluazuron was introduced in Australia in 1994, but shortly afterwards the registrant voluntarily stopped sales until import tolerances were approved in major countries importing beef from Australia. It was re-introduced in 1998. Thus it took 12 years for the first resistance case to be reported. In Brazil, there was no interruption in the use of fluazuron, ticks of the Jaguar strain used for the study were collected in 2011, and at the affected farm, reduced control after fluazuron treatment had already been observed in 2010 [241], i.e. about 16 years after regular use of fluazuron in the country.

Reck et al. [241], have also reviewed the time it took for *R. microplus* resistance to appear to several chemical classes in Australia and Brazil. Amitraz resistance took four years to develop in Australia (introduced in 1977; first resistance report in 1981), and 16 years in Brazil (introduced in 1977; first resistance report in 1993). Resistance to synthetic pyrethroids took 7–8 years to develop, both in Australia and Brazil (introduced in the early 1980s, first resistance reports in 1989 in both countries). Resistance to macrocyclic lactones took about 20 years to develop in Brazil (introduced in 1996; first resistance report in Brazil in 2001), whereby use against *R. microplus* became popular only after the introduction of long-acting formulations in the 1990s (ivermectin slow-release bolus, 3.15% injectables, etc.). *R. microplus* resistance to macrocyclic lactones has not yet been reported in Australia. Resistance to fipronil took eight years to develop in Brazil (introduced in 1996, first resistance report in 2004). Fipronil is not approved for use on cattle in Australia. In this context, the appearance of fluazuron resistant *R. microplus* ticks within 12–16 years after introduction is comparable with other chemical classes.

*R. microplus* resistance to fluazuron seems still to be rather limited in Brazil, but spreading. A study in 2012 in the North-Central region of Bahia State did not find resistance or tolerance (i.e. low resistance with RR of 3–5) to fluazuron in *R. microplus* field populations collected in seven different municipalities [240]. A literature review on *R. microplus* resistance in Brazil from 2015 [135] reports the previously mentioned investigation [241] as the only case of fluazuron resistance described so far. However, a field study in Brazil in 2016 [192] reports resistance or reduced susceptibility to fluazuron in four (two in the Southeast and two in the Mid-West of Brazil) of 27 properties investigated. Other studies in Colombia in 2015 [235] and Uruguay in 2014 and 2017 [62, 63] did not find resistance to fluazuron in local populations of *R. microplus*. We have not found published reports on fluazuron resistance in further Latin American countries.

### Lufenuron

Resistance to lufenuron has been reported in laboratory strains and field populations of several agricultural pests, [230]. So far, no confirmed reports on resistance of fleas to lufenuron have been published, almost three decades after its introduction for flea control in pets. This is in spite of the fact that the cat flea (*C. felis*), the main species infesting pets worldwide, had previously developed resistance to numerous insecticides including organochlorines, organophosphates, carbamates and synthetic pyrethroids [23]. However, these resistances seem to be not as strong as in other arthropod pests: RRs reported are usually < 50, except for malathion, for which an RR of 690 has been reported [23].

As mentioned earlier, the market dynamics of lufenuron resulted in lower usage and consequently in lower selection pressure at a global scale, which can partially explain the absence of resistance so far. Resistance was reported to fipronil already in 2001 (cited by [52]), but not to pyriprole, a more recent compound from this class. However, we have not found later records of additional cases of flea resistance to fipronil in spite of its massive use worldwide. To our knowledge, resistance of *C. felis* has not been reported to any of the other new classes of insecticides introduced for on-animal flea control since about 1990 [251]. In addition to BPUs (lufenuron), this includes neonicotinoids (imidacloprid, nitenpyram, dinotefuran), macrocyclic lactones (selamectin), juvenile-hormone analogues (pyriproxyfen, methoprene), spinosyns (spinosad, spinetoram) [19, 262], oxadiazines (indoxacarb) [84], and the isoxazolines afoxolaner [261], fluralaner [247] and sarolaner [201]. This has resulted in an unprecedented situation in terms of number of chemical classes with different mechanisms of action available for the control of a single veterinary parasite. In addition, several products have also become available with mixtures of two or more active ingredients affecting fleas through different mechanisms of action [18]. This development reflects the competition among manufacturers eager to get a share of this largest parasiticidal market. An indirect consequence of this development is a very favorable situation in terms of resistance prevention and management. If a pet owner perceives a given product to be ineffective and tries another one, the availability of so many and such powerful flea control products makes it likely that a product with another mechanism of action is selected, even without knowing it. These and other factors (e.g. the huge reservoir of susceptible fleas in untreated pets and wild carnivores) may have contributed to preventing resistance development in the cat flea to lufenuron and other compounds during the last few years and may help to prevent it in the future.

### Teflubenzuron

Resistance to teflubenzuron has been reported for several agricultural pests [230]. Sea lice developed resistance against various parasiticides, e.g. against organophosphates (dichlorvos and azamethiphos) in Norway, Scotland and Canada, and against synthetic pyrethroids (deltamethrin) in Norway, already in the 1990s [78, 236]. Later on, resistance to macrocyclic lactones (emamectin benzoate) was reported in Canada, Europe and Chile [146]. We have not found reports on resistance of *L. salmonis* or other sea lice species to teflubenzuron in the scientific literature. This may be related to the fact that teflubenzuron-based products were not approved in all countries relevant for salmon farming, and where approved, their use was rather modest compared with other alternative active ingredients with adulticidal effect, and in some countries quantity of use is restricted by regulators depending on the outcome of site-specific environmental modeling (e.g. in Scotland) [259]. The same applies to diflubenzuron. This modest use suggests a rather low selective pressure for BPU resistance on sea lice, which may explain the absence of confirmed cases of resistance to this chemical class.

### Triflumuron

According to a recent review [230], resistance to triflumuron has been reported in several countries in agricultural pests and houseflies. Regarding resistance of veterinary parasites to triflumuron, first reports on sheep body lice resistance in Australia were already published in 2005 [249]. Later on, resistance was confirmed in laboratory assays conducted on several field strains collected from sheep farms and showed RRs ranging from 5.7 to 93.8 and clear side-resistance with diflubenzuron [153]. So far, louse resistance to triflumuron seems not to be present in New Zealand [132].

Laboratory assays on larvae of blowflies (*L. cuprina* and *L. sericata*) collected from farms in New Zealand in the years 2010–2011 identified field strains of both species with decreased susceptibility to triflumuron with one *L. sericata* strain showing an RR of >14,000 and several other *L. sericata* strains with RR > 1000. The highest RR determined for *L. cuprina* strains was 16 [284].

Considering that triflumuron was introduced for louse control in Australia in 1993 [141] and at about the same time in New Zealand (also for blowfly strike control), it took about 12 years for the first confirmed cases of sheep body louse resistance to triflumuron to be reported in Australia, and about 17 years for the first cases of reduced blowfly susceptibility to triflumuron in New Zealand.

### Novaluron

So far, there are no reports on resistance of fleas or cattle ticks to novaluron, which is not surprising considering its recent introduction and limited use against these pests.

## Perspectives and outlook

In the following section, we comment briefly on some potential commercial uses of BPUs against veterinary parasites that have been poorly or not investigated at all so far, as well as on possible extensions of their use to other countries where they are currently not approved for certain veterinary uses. We also briefly discuss the future risks for resistance development where it has not yet appeared, or where it is spreading and increasing when already present.

### Commercial use

Since their discovery about 40 years ago, BPUs have been intensively investigated for their potential as veterinary parasiticides, and four decades later it would seem unlikely that unexploited opportunities remain undiscovered that could lead to new veterinary medicines.

However, some approved uses of BPUs have been poorly exploited, e.g. horn fly control in cattle. The main reason is insufficient commercial attractiveness when compared with available alternatives, as previously discussed. Horn fly control in most of the Americas and elsewhere is increasingly relying on active ingredients of few chemical classes, mainly organophosphates, synthetic pyrethroids, fipronil and macrocyclic lactones. Field resistance of horn flies to synthetic pyrethroids is widespread and often very high, and resistance to organophosphates is not unusual [100]. Ivermectin resistance has been demonstrated in laboratory strains [32], and it is probably only a matter of time for resistance to fipronil to develop where it is currently used. To our knowledge, an approach to horn fly control with BPUs targeting the adult flies instead of the larvae in the cowpats has not been commercially explored, although a strong chemosterilant effect after topical treatment of cattle with diflubenzuron was reported early by Kunz et al. [172, 173]. An approach to systemically chemosterilize the adult flies, i.e. through the host’s blood has not been investigated either. Horn flies are known to remain for long periods of time on their hosts, very much like fleas on dogs and cats, and cattle ticks (*R. microplus*) on cattle. Due to its specific properties, diflubenzuron is not the right candidate for trying such a systemic approach to horn fly control: it is poorly absorbed, and what is absorbed is quickly metabolized or excreted. Fluazuron is not appropriate either, because it is only effective against ticks. But lufenuron looks theoretically appropriate: it is highly effective as an insect chemosterilant [230], has a systemic mode of action, and a single treatment may ensure efficacy for months due to its ability to be stored in the host’s fat as shown to happen in cats against fleas [116]. A comparable situation exists regarding other blood-sucking parasites of livestock that spend most of their life cycle on their hosts, e.g. several biting lice species or sheep keds (*Melophagus ovinus*). A major hurdle to this approach could be excessive residues, although withholding periods of up to four months are currently quite common for high concentration ivermectin injectables.

Another remarkable gap regarding exploration of the veterinary potential of BPUs is the field of myiasis that affect livestock other than sheep blowflies, particularly screwworms (e.g. *Cochliomyia hominivorax*), warble flies (*Hypoderma* spp.), human bot flies (*Dermatobia hominis*) and nasal bot flies (*Oestrus ovis*). When fed to adult *C. hominivorax* flies, diflubenzuron reduced egg hatching of oviposited eggs [60]. Successful off-label use of lufenuron to prevent *D. hominis* and *C. hominivorax* myiases has been reported in dogs in Brazil [58]. We have not found other reports exploring such potential uses. In all these myiases, the whole larval life cycle is completed within the host’s body and remains exposed for weeks and even months to any chemical circulating in the bloodstream or deposited in the tissues. Among those BPUs already used as veterinary medicines, lufenuron and perhaps also novaluron could theoretically be effective against these species. During pre-clinical development, lufenuron showed high efficacy against larvae of other Dipteran myiases such as *L. cuprina* and *L. sericata* (P. Junquera, unpublished results) and it has a systemic mode of action. The reasons why lufenuron has not been explored against these myiases is probably lack of commercial interest, possibly related to the fact that macrocyclic lactones are very effectively used for the control and prevention of these myiases. As previously mentioned, excessive residues in food-producing animals could act as a limiting factor for such uses.

The global perspective reveals another possible opportunity: whereas triflumuron and diflubenzuron are very successfully used against the sheep body louse in Australia and New Zealand, neither compound is used for on-animal control of lice on sheep in Europe or Latin America, where sheep body lice are also an important pest and sheep lousicides still make up an important share of the parasiticides market. It is not clear whether this is due to lack of commercial interest for manufacturers or to regulatory constraints. In both regions, synthetic pyrethroids are still the most widely used chemical class for louse control. In Argentina, no cases of louse resistance have been reported [222]. In the UK, reliable laboratory and field data indicated possible resistance to deltamethrin in 2001 [100] but we have not found confirming reports since then. Regardless of the reasons, should louse resistance to synthetic pyrethroids become a problem in Europe or Latin America, BPUs could become a valid alternative. A comparable opportunity can develop regarding blowfly strike prevention with diflubenzuron (or other BPUs) in Europe. Blowfly strike, mainly by *L. sericata,* is a serious disease in the UK, Ireland and the Netherlands but BPUs have not been registered for this use in the EU.

During pre-clinical development, fluazuron showed high topical efficacy against the red fowl mite, *Dermanyssus gallinae* (Junquera, unpublished results), a blood sucking mite that is a serious pest to laying hens worldwide. To our knowledge, no further studies have been conducted to investigate this potential. However, these mites have developed resistance to most products currently used for their control [2, 47] and new active ingredients without cross-resistance are urgently needed. On-animal administration is probably not practicable because it is likely to leave excessive residues in the eggs. But adequate treatment of the mites’ habitat off-the animals could perhaps provide sufficient control of the mite populations.

A global look over BPUs also shows that only six compounds are commercially used against veterinary parasites, whereas several other BPUs commercialized against agricultural or forestry pests have not yet found a veterinary use, e.g. chlorfluazuron (Ishihara, discovered in 1983), flufenoxuron (Shell, discovered in 1987), hexaflumuron (Dow Elanco, discovered in 1984), or noviflumuron (Dow Agro, discovered in 2001) [83]. Chlorfluazuron has been reported to be effective against larvae of the cat flea (*C. felis*) after exposure to treated larval medium [252]. We have not found additional evidence in the literature that these compounds have been investigated for their potential against other veterinary parasiticides although there has been patent activity and occasional conference posters indicating the potential use of hexaflumuron against sea lice. Recent information regarding a recommended MRL for hexaflumuron in finfish suggests that it may be in development for sea louse control [95].

A reason may be that the companies that discovered and/or marketed these compounds first were not active or not interested in the market for veterinary parasiticides, which is substantially smaller than the crop protection market. Increased availability after expiry of patent protections makes it easier for more companies to explore new potentials, also in animal health. The introduction of diflubenzuron for louse and blowfly control on sheep by Hoechst in 1993 in Australia and New Zealand, and the introduction of teflubenzuron for sea lice control by Trouw in Norway in 1996 seem to be examples of this.

### Resistance

As previously described, resistance of veterinary parasites to BPUs has evolved differently. Resistance of sheep blowflies to diflubenzuron developed very fast and became very high in Australia, which led to discontinuing its approval for blowfly control in 2008, 15 years after its introduction. In fact, resistance was already there at introduction in the form of cross-resistance with organophosphates, as previously explained (see Section Diflubenzuron). Resistance of sheep body lice to both triflumuron and diflubenzuron took about 12 years to appear in Australia and resistance to fluazuron took about the same time to develop in Australia and 16 years in Brazil. However, these cases are not yet comparable with the dramatic situation that led to the discontinuing of diflubenzuron in Australia for blowfly strike control. No resistance has been reported yet to diflubenzuron in horn flies, to lufenuron in fleas, or to diflubenzuron, teflubenzuron or lufenuron in sea lice.

The global perspective confirms what is usually experienced elsewhere regarding resistance development of parasites to parasiticides: the more intensively and extensively a chemical class is used, the higher the selection pressure, the faster resistance develops and the stronger it becomes. A substantial factor that enhances or diminishes selection pressure is the availability of refuges for the parasites, i.e. hosts that are not treated with parasiticides, mostly wildlife. From this perspective, the outlook for those BPUs used as veterinary parasiticides is also different.

Regarding horn flies on cattle, as long as BPUs are so scarcely used as until now, it is unlikely that this parasite will develop resistance to diflubenzuron: selection pressure is very low, even though horn flies are very cattle specific and refuges are often very scarce in many cattle properties. However, should usage of diflubenzuron or another BPU against horn flies increase in regions with a history of strong organophosphate resistance, cross-resistance to BPUs may be found, as it happened with sheep blowflies.

The risk that dog and cat fleas develop resistance to lufenuron is also low: it is moderately used (i.e. many dogs and cats are not treated with it), many alternative products with other mechanisms of action and no cross-resistance are available, and fleas are not host-specific and may find alternative hosts as refuges (small rodents, foxes, etc.). This results in low selection pressure.

The risk for the appearance of sea lice resistance to diflubenzuron, teflubenzuron or lufenuron depends strongly on their future use as well. Until now the usage of diflubenzuron and teflubenzuron has been modest and in two waves: from about 1996 to 2000, and from 2009 until now, with a long gap in between [78, 97], i.e. there was probably not a strong selection pressure. The same applies to lufenuron that has just been introduced in this market. However, effective alternatives are now scarce due to increasing resistance to other compounds [1], which may result in increasing reliance on BPUs with the corresponding increase of the selection pressure. The salmonid aquaculture industry is working to adapt and implement management practices from land-based integrated pest management (IPM), including the development of non-medicinal delousing techniques and prevention strategies (A. Macdonald, personal communication).

The situation is more worrying for fluazuron against cattle ticks. Resistance to old classic acaricides is very frequent in many countries, e.g. in Brazil [136], Colombia [235] or Mexico [246] and control relies more and more on fluazuron and macrocyclic lactones, in Latin America also on fipronil. However, resistance to fipronil and the macrocyclic lactones is quickly increasing. In Brazil, out of 104 cattle tick field samples collected in Rio Grande do Sul, about 60% showed resistance to ivermectin, and about 54% to fipronil, with a significant number of multi-resistant field strains [165]. A similar situation has been reported for Uruguay [62, 63]. Reliance on these compounds is already excessive and will increase the selection pressure. In addition, *R. microplus* is highly cattle-specific and in most cattle properties very few alternative hosts can serve as refuges, if at all. Moreover, vehicles to create additional refugial tick populations on-farm as done for ruminant nematodes in some countries [82] needs to be fully investigated. As a consequence spreading and strengthening of resistance seems unavoidable unless recommended IPM approaches [100, 122, 217] are implemented or new chemical classes of tickicides with new modes of action are introduced.

Regarding body louse control on sheep in Australia and New Zealand, control has relied strongly on triflumuron and diflubenzuron in the last decades, but now the use of spinosad (from Elanco) and imidacloprid (from Bayer) is increasing. However, *B. ovis* is also very host-specific and refuges (e.g. stray sheep) are almost non-existent on most properties. Therefore, implementation of IPM approaches as already proposed by several authors [100, 152] should not be delayed.
